# Structure of nascent 5S RNPs at the crossroad between ribosome assembly and MDM2–p53 pathways

**DOI:** 10.1038/s41594-023-01006-7

**Published:** 2023-06-08

**Authors:** Nestor Miguel Castillo Duque de Estrada, Matthias Thoms, Dirk Flemming, Henrik M. Hammaren, Robert Buschauer, Michael Ameismeier, Jochen Baßler, Martin Beck, Roland Beckmann, Ed Hurt

**Affiliations:** 1 Heidelberg University Biochemistry Center (BZH), Heidelberg, Germany; 2 Gene Center, Ludwig-Maximilians-Universität München, Munich, Germany; 3 European Molecular Biology Laboratory (EMBL), Heidelberg, Germany; 4 Max Planck Institute of Biophysics, Frankfurt am Main, Germany

**Keywords:** Electron microscopy, Proteins, Ribosome, Translation, Cryoelectron microscopy

## Abstract

The 5S ribonucleoprotein (RNP) is assembled from its three components (5S rRNA, Rpl5/uL18 and Rpl11/uL5) before being incorporated into the pre-60S subunit. However, when ribosome synthesis is disturbed, a free 5S RNP can enter the MDM2–p53 pathway to regulate cell cycle and apoptotic signaling. Here we reconstitute and determine the cryo-electron microscopy structure of the conserved hexameric 5S RNP with fungal or human factors. This reveals how the nascent 5S rRNA associates with the initial nuclear import complex Syo1–uL18–uL5 and, upon further recruitment of the nucleolar factors Rpf2 and Rrs1, develops into the 5S RNP precursor that can assemble into the pre-ribosome. In addition, we elucidate the structure of another 5S RNP intermediate, carrying the human ubiquitin ligase Mdm2, which unravels how this enzyme can be sequestered from its target substrate p53. Our data provide molecular insight into how the 5S RNP can mediate between ribosome biogenesis and cell proliferation.

## Abstract (web summary)

The structure of the assembly-competent 5S RNP was elucidated by cryo-EM. These findings provide molecular insight into how this module incorporates into the nascent pre-60S ribosome and how it can affect the MDM2–p53 pathway in human cells.

## Main

Eukaryotic ribosome biogenesis is a very energy-consuming process, during which ~80 ribosomal proteins and 4 ribosomal RNAs (28S/25S, 18S, 5.8S and 5S rRNA) assemble along a complicated pathway that starts in the nucleolus and ends in the cytoplasm^1^. This process is driven by approximately 200 ribosome assembly factors, which transiently associate with the developing pre-ribosomes. Three of the ribosomal RNAs, 18S, 5.8S and 25S/28S rRNA, are synthesized by RNA polymerase I and are initially part of a large precursor (35S pre-rRNA in yeast and 47S pre-rRNA in human) before the mature rRNAs are released through RNA processing and trimming^1,2^. The 5S rRNA is separately transcribed by RNA polymerase III and, after joining with its cognate ribosomal proteins uL18 and uL5 (previously called Rpl5 and Rpl11, respectively^3^), forms the minimal trimeric 5S RNP core complex, which is eventually incorporated into the pre-60S particle^4,5^. Assembly of the 5S RNP and its subsequent incorporation into the pre-ribosome are not well understood, but two additional pre-60S factors, Rpf2 (BXDC1 in human) and Rrs1 (RRS1 in human), are known to be involved in both yeast and human cells^2,6–9^. It is clear that within pre-60S particles in yeast, the Rpf2–Rrs1 heterodimer binds to the 5S rRNA and uL18 at the base of the central protuberance, whereas their carboxy-terminal extensions penetrating into the pre-60S core make contact with distinct 25S rRNA helices (H80–H88 of domain V) and a number of 60S biogenesis factors (for example Nog2/Nug2)^10^. Thus, the Rpf2–Rrs1 heterodimer apparently helps anchor the 5S RNP in a premature 180°-twisted orientation onto the pre-ribosome through their direct contact and is further involved in coordinating 5S RNP rotation with maturation of functional centers during large ribosomal subunit assembly^7–9,11–13^. The release of this heterodimer is coupled with the 5S RNP relocation to its final position during 60S biogenesis^14^. A similar structural rearrangement may also take place during human ribosome assembly, but has not yet been demonstrated^2^.

Another factor playing a role in 5S RNP biogenesis is symportin Syo1 (HEATR3 in human), which synchronizes the nuclear transport of uL18 and uL5 by forming a Syo1–uL18–uL5 import complex that recruits the karyopherin Kap104, using Syo1’s amino-terminal PY-NLS^15,16^. What happens after nuclear import is unclear, but it is postulated that following the RanGTP-mediated release of Kap104 from the Syo1–uL18–uL5 import complex, the newly transcribed 5S rRNA binds to uL18–uL5, forming the 5S RNP that subsequently assembles into the pre-ribosome^17^.

As ribosome biogenesis is tightly regulated and controlled for the quality and stability of nascent ribosomes, its status is constantly communicated to other cellular pathways, such as gene expression, cell cycle progression, protein and RNA turnover and nutrient availability^4,18^. Here, one important sensor is the free pool of 5S RNP that accumulates upon inefficient incorporation into pre-ribosomes due to, for example, inhibition of rRNA synthesis, mutations or haploinsufficiency of ribosomal proteins. The pool of free 5S RNP can thus be used by the cell as a proxy for compromised ribosomal biogenesis, triggering, for example, the MDM2–p53 pathway^19,20^. It has been suggested that either the free 5S RNP or individual ribosomal proteins, most prominently Rpl11/uL5, recruit the ubiquitin ligase Mdm2, which reduces ubiquitination of p53, thereby stabilizing p53, with all of the known consequences for health and disease^21,22^. However, the structural details underlying this coordinated process remained largely unknown.

In this study, we set out to examine the mechanism of 5S RNP formation and its subsequent assembly into pre-ribosomes. We reconstituted the assembly-competent 5S RNP, which is a conserved hexameric complex, composed of Syo1, Rpf2, Rrs1, uL18, uL5 and 5S rRNA, and solved its structure by cryo-electron microscopy (cryo-EM). Moreover, we could reconstitute a simpler 5S RNP complex, comprising the human factors uL18 and uL5, but instead of Syo1 and Rpf2–Rrs1, it carries the ubiquitin ligase Mdm2, which explains how a ribosome assembly intermediate can sequester this ubiquitinating enzyme from its substrate, p53.

## Results

### Identification of the conserved hexameric 5S RNP precursor

**Fig. 1:**
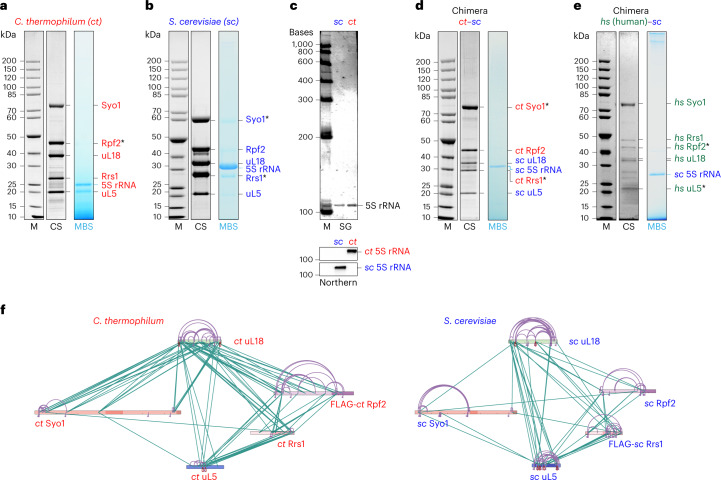
Isolation of hexameric 5S RNP complexes from *Chaetomium thermophilum* and *Saccharomyces cerevisiae* and reconstitution of thermophile–yeast and human–yeast 5S RNP chimeras. **a**, Affinity purification of *C. thermophilum* (*ct*) 5S RNP via *ct*Rpf2. **b**, Split-tag affinity purification of *S. cerevisiae* (*sc*) 5S RNP via *sc*Syo1–*sc*Rrs1 pair. **c**, SYBR Green II staining (SG) to detect the total RNA and northern blot analysis (Northern) of *ct* and *sc* 5S rRNA extracted from the isolated 5S RNP complexes and probed with *sc*-specific and *ct*-specific 5S rRNA oligonucleotide probes. **d**, Split-tag affinity purification of the thermophile–yeast 5S RNP (*ct*–*sc* chimera) via *ct*Syo1–*ct*Rrs1 pair. **e**, Split-tag affinity purification of the human–yeast 5S RNP (*hs*–*sc* chimera) via *hs*uL5–*hs*Rpf2 pair. The final eluates from **a**, **b**, **d** and **e** were analyzed by SDS–PAGE and Coomassie staining (CS). Labeled bands were identified by mass spectrometry or by methylene blue staining (MBS) to reveal the 5S rRNA. One caveat of SDS–PAGE/MBS staining is that the structured 5S RNA may not be fully denatured by SDS, causing different running behavior. To correctly analyze the 5S RNA, we also performed denaturing urea PAGE of the 5S RNP samples from *sc* and *ct* (see Fig. 1c). A protein molecular-weight marker standard (M) is shown on the left for the SDS–PAGE gels (**a**,**b**,**d**,**e**). An RNA molecular-weight standard (indicated in bases) is shown for the urea PAGE gel (**c**). Asterisks indicate the baits used for each affinity purification step. All purifications were done at least twice with a similar outcome. **f**, XL-MS of the affinity-purified 5S RNP from *C. thermophilum* (left) and *S. cerevisiae* (right) using DSS-H12. The protein primary structures of Syo1, uL18, uL5, Rpf2 and Rrs1 are shown, and specific regions are indicated in darker colors. Intermolecular crosslinks are shown in green, and intramolecular crosslinks are shown in purple. The xiNET tool^45^ was used for visualization. Source data

**Fig. 2:**
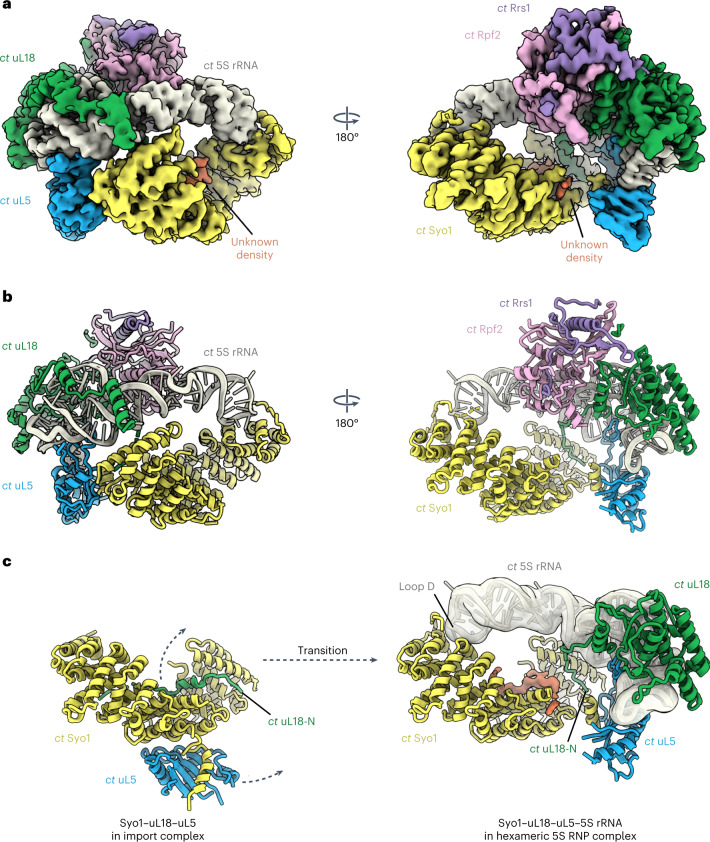
Cryo-EM structure of the conserved 5S RNP. **a**,**b**, Cryo-EM map (**a**) and model (**b**) of the *C. thermophilum* 5S RNP (for purification, see Fig. 1a). The subunits of the 5S RNP are shown in different colors and labeled. **c**, Rearrangement of Syo1, uL5 and uL18-N before (left; PDB ID 5AFF) and after incorporation into the 5S RNP complex.

**Extended Data Fig. 1:**
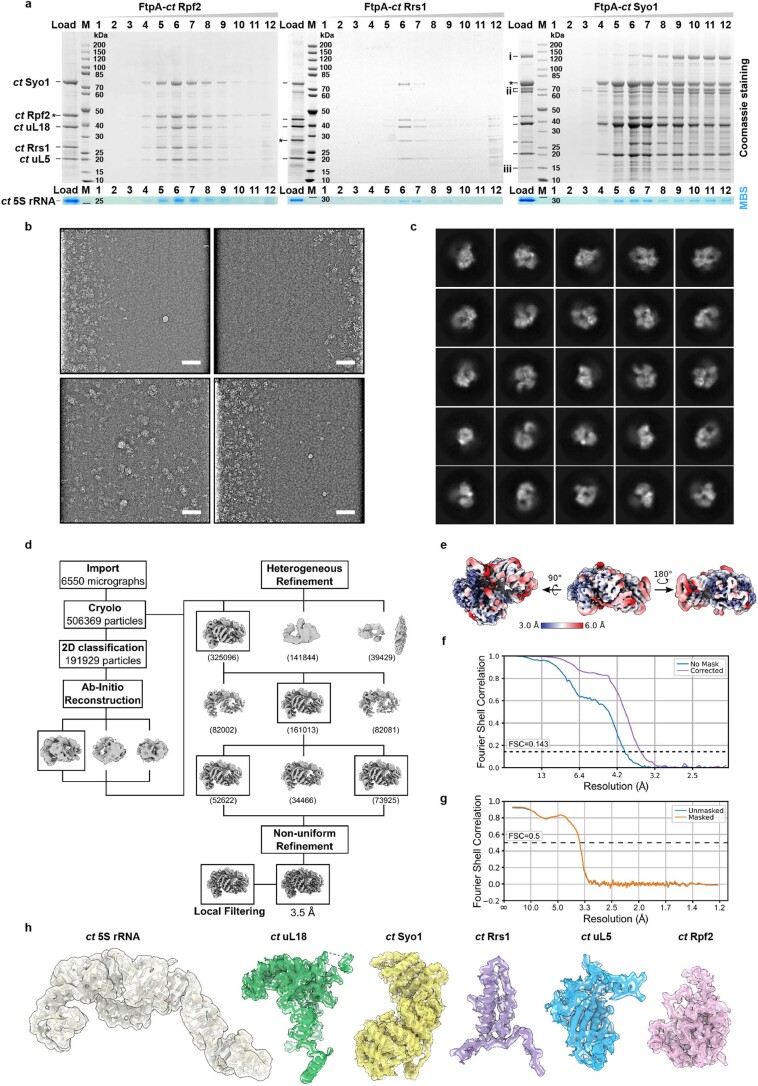
Purification and cryo-EM analysis of the hexameric 5S RNP complex isolated directly from Chaetomium thermophilum. **a**, Affinity purification of FLAG-TEV-ProtA (FtpA)-tagged *ct*Rpf2 (left), *ct*Rrs1 (middle) and *ct*Syo1 (right) from *C. thermophilum*. The final FLAG eluates (Load) were separated by sucrose gradient centrifugation, and fractions were analyzed by SDS-PAGE. Bait proteins are labeled with asterisks. M: Molecular weight marker. The hexameric Syo1–uL18–uL5–Rpf2–Rrs1-5S rRNA pulled down by these different baits is typically recovered in fractions 6-7. A simpler Syo1–uL18–uL5-5S rRNA complex is also detected in fractions 4-5 of the FtpA-*ct*Syo1 purification. Presence of 5S rRNA was identified by methylene blue staining (MBS). The labeled proteins were identified by mass spectrometry: *ct*Syo1 (CTHT_0033460), *ct*Rpf2 (CTHT_0061110), *ct*Rrs1 (CTHT_0057260), *ct*uL18 (CTHT_0063640), and *ct*uL5 (CTHT_0072970); additional proteins in the FtpA-*ct*Syo1 purification were identified as (i) CTHT_0061210, (ii) CTHT_ 0056370 (Fpr4 homologue) and (iii) CTHT_0025560. These purifications have been done at least 3 times with similar outcome. **b**,**c**, Cryo-EM representative micrographs of the ct 5S RNP low-pass filtered to 20 Å, displayed with inverted contrast (out of 6550 micrographs used for processing) (**b**) and selected 2D classification averages (**c**). Scale bar: 50 nm. **d**, Cryo-EM processing scheme. The overall resolution of the final 3D reconstruction was 3.5 Å. **e**, Final 3D reconstruction of the *ct* 5S RNP filtered and colored according to local resolution. **f**, Fourier shell correlation (FSC) curves of the final structure showing unmasked (No mask) and masked (Corrected) FSC curves. **g**, Model-to-map FSC curve. **h**, Segmented cryo-EM densities and models of the individual subunits of the *ct* 5S RNP. Source data

To study the formation of 5S RNP and its incorporation into pre-60S particles, we affinity purified Rpf2 and Rrs1 directly from the thermophilic eukaryote *Chaetomium thermophilum* (*ct*), which was previously used to solve 90S pre-ribosome structures^23^. However, in contrast to the findings in yeast, neither of these factors co-enriched pre-60S particles. Instead, they co-precipitated a free hexameric 5S RNP composed of the 5S rRNA, uL18 and uL5, the Rpf2–Rrs1 heterodimer^7–9^, and unexpectedly Syol, the common nuclear import adaptor of uL18 and uL5 (Fig. 1a,c and Extended Data Fig. 1a). These six components co-migrated as a complex on density gradients and formed discrete particles, which were imaged by negative-stain EM and solved by cryo-EM (Fig. 2, Extended Data Fig. 1 and Table 1).

**Table 1:** Data collection, refinement and model statistics

	Hexameric *ct*5S RNP (EMD-13134) (PDB 7OZS)	Hexameric *ct*–*sc* 5S RNP monomer (EMD-16037)	Hexameric *ct*–*sc* 5S RNP dimer (EMD-16038)	Human MDM2–5S RNP (EMD-16036) (PDB 8BGU)	Pre-60S with human 5S RNP (EMD-16040)
**Data collection and processing**					
Camera	Gatan K2 Summit	Gatan K2 Summit	Gatan K2 Summit	Gatan K2 Summit	Falcon II
Magnification	130,000	130,000	130,000	130,000	75,000
Voltage (kV)	300	300	300	300	300
Electron exposure (e^−^ Å^−2^)	43.6	46	46	38	25
Defocus range (μm)	0.4–4.0	0.5–2.8	0.5–2.8	0.4–4.0	0.65–3.7
Pixel size (Å)	1.059	1.059	1.059	1.059	1.084
Symmetry imposed	C1	C1	C2	C1	C1
Micrographs collected (no.)	7,040	2,119	2,119	2,504	4,660
Initial particle images (no.)	506,369	257,706	257,706	943,595	265,032
Final particle images (no.)	126,547	56,245	68,563	219,620	34,299
Map resolution (Å)	3.5	4.1	4.0	4.1	3.7
FSC threshold	0.143	0.143	0.143	0.143	0.143
**Refinement**					
Model resolution (Å)	3.6			4.5	
FSC threshold	0.5			0.5	
Map sharpening B-factor (Å^2^)	−80			−80	
Model composition					
Non-hydrogen atoms	12,851			4,779	
Protein residues	1,337			447	
Nucleotide residues	119			121	
Ligands	0			1	
R.m.s deviations					
Bond length (Å)	0.005			0.003	
Bond angle (°)	1.034			0.837	
**Validation**					
Molprobity score	1.42			1.54	
Clash score	3.82			4.09	
Poor rotamers (%)	0.28			0.00	
Ramachandran plot					
Favored (%)	96.32			95.01	
Allowed (%)	3.68			4.99	
Disallowed (%)	0.00			0.00	
Map versus model correlation coefficient (mask)	0.85			0.86	

To investigate whether this hexameric 5S RNP intermediate is also formed in mesophilic organisms, we switched to the powerful genetic system of *Saccharomyces cerevisiae* (*sc*), where eukaryotic ribosome biogenesis has been best characterized. A similar yeast 5S RNP precursor could be assembled by co-overexpression of the orthologous yeast factors followed by Syo1–Rrs1 split-tag affinity purification. The *S. cerevisiae* 5S RNP also formed a hexamer, again composed of 5S rRNA and associated uL18, uL5, Rpf2, Rrs1 and Syo1 in approximately stoichiometric ratios (Fig. 1b,c). This hexameric 5S RNP might also exist as a free pool in yeast under normal (that is, non-overproducing) growth conditions but in low concentration due to its efficient incorporation into pre-60S particles^24^. In this respect, yeast differs from *C. thermophilum* and also from human cells^25^, in which a substantial amount of the 5S RNP exists as a free pool.

**Extended Data Fig. 2:**
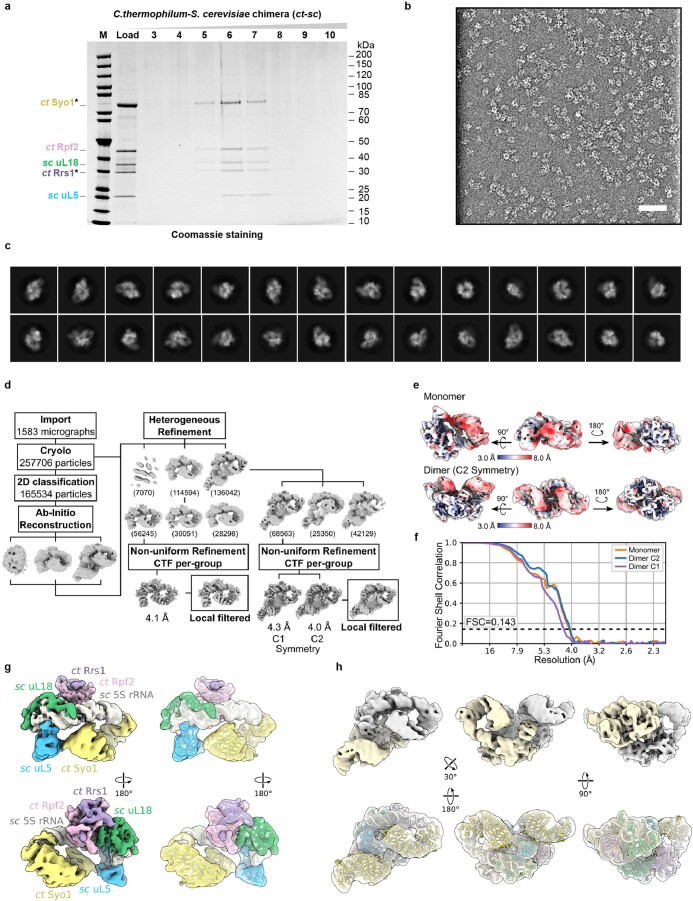
Purification, cryo-EM analysis and structure of the ct–sc 5S RNP chimera. **a**, Split-tag affinity purification of the ct–sc 5S RNP chimera from yeast cells via ProtA-TEV-ctSyo1 and FLAG-ctRrs1 under conditions of ctRpf2 co-expression. The final FLAG eluate (Load) was separated by sucrose gradient centrifugation, and fractions were analyzed by SDS-PAGE. Bait proteins are labeled with asterisks. M: Molecular weight marker. Labeled bands were identified by MS. This purification has been done at least 5 times with similar results. **b**, **c**, Cryo-EM representative micrograph of the ct–sc 5S RNP chimera low-pass filtered to 20 Å, displayed with inverted contrast (out of 1583 micrographs used for processing) (b) and selected 2D classification averages (c). **d**, Cryo-EM processing scheme. The overall resolution of the final 3D reconstructions was 4.1 Å for the monomeric 5S RNP and 4.3 Å/4.0 Å for the dimeric 5S RNP without/with application of C2 symmetry. **e**, Final 3D reconstructions filtered and colored according to local resolutions. **f**, FSC curves of the final 3D reconstructions (masked). **g**, **h**, Cryo-EM densities and fitted models of the ct–sc 5S RNP assembled with C. thermophilum and S. cerevisiae factors for the monomeric (g) and dimeric (h) forms. 5S RNP factors are labeled in (g).

Based on our findings obtained from the two fungal model systems, we were also able to isolate a chimeric *ct–sc* 5S RNP hexamer, which was achieved by the co-overexpression of *C. thermophilum* Rpf2, Rrs1 and Syo1 in yeast. The 5S RNP chimera obtained after split-tag affinity purification of *ct*Syo1 and *ct*Rrs1 contained endogenous yeast 5S rRNA, uL18 and uL5 together with the thermophile factors Rpf2, Rrs1 and Syo1 (Fig. 1d and Extended Data Fig. 2).

### Assembly of human 5S RNP into yeast pre-60S particles

**Extended Data Fig. 3:**
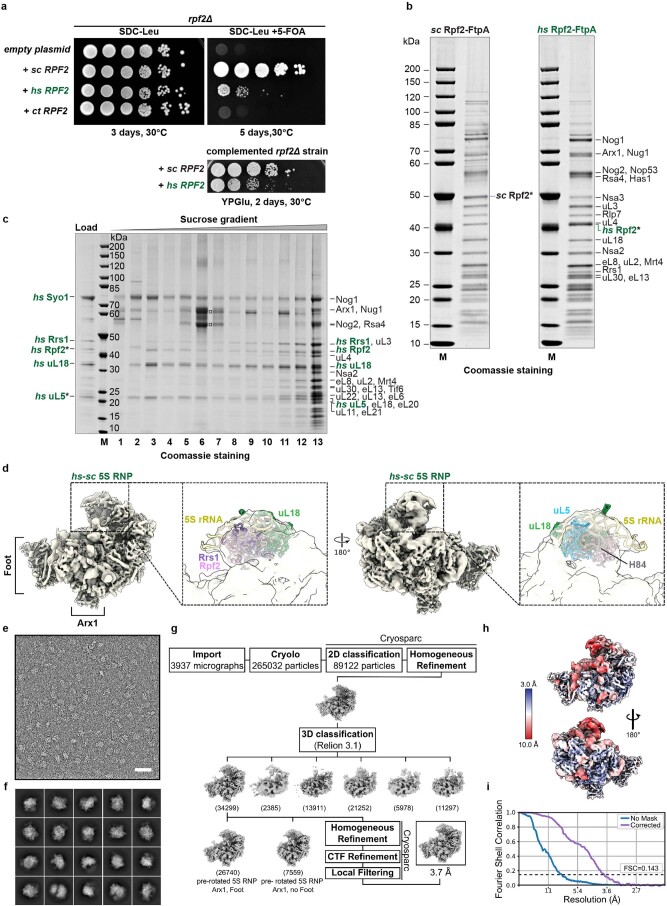
Cryo-EM analysis of the human–yeast 5S RNP chimera within yeast pre-60S particles. **a**, Growth analysis of the nonviable yeast rpf2Δ strain complemented by hsRpf2 but not ctRpf2 on 5-FOA plates (upper panel) and YPGlu-plates (lower panel). To test for complementation, yeast rpf2Δ or rrs1Δ strains carrying RPF2 or RRS1 in a URA3-containing plasmid were transformed with plasmids carrying scRPF2, hsRPF2, ctRPF2, scRRS1, hsRRS1 or ctRRS1, and grown on selective SDC-Leu or SDC-Leu + 5-FOA plates for 3 or 5 days at 30 °C. **b**, Affinity purification of yeast Rpf2-FtpA or human Rpf2-FtpA from the complemented yeast rpf2Δ strain (growth analysis is shown in panel a), yielding pre-60S particles with a similar composition. Bait proteins are labeled with asterisks. Purifications have been done twice with similar results. **c**, Sucrose gradient centrifugation of the final eluate from the hs–sc 5S RNP chimera obtained from yeast co-overexpressing hsuL18, hsRrs1 and hsSyo1 and baits hsuL5 and hsRpf2 (labeled with asterisks). The free 5S RNP was detected in fraction 3 and pre-60S particles containing the human 5S RNP factors in fraction 13. Labeled bands were identified by mass spectrometry. The minor band above hsuL18 is hsuL18 with small amounts of scuL18. The two intense bands in fraction 6 (□) were human keratin contaminants. This purification has been done twice with similar results. **d**, Cryo-EM structure of the yeast pre-60S particle carrying hsRpf2, hsRrs1, hsuL18, and hsuL5 (panel C, fraction 13), which, together with the sc 5S rRNA, are shown in different colors. **e**, **f**, Cryo-EM micrograph of human 5S RNP assembled into yeast pre-60S, low-pass filtered to 20 Å and displayed with inverted contrast (out of 3937 micrographs used for processing) (e) and selected 2D classification averages (f). Scale bar: 50 nm. **g**, Cryo-EM processing scheme. The overall resolution was 3.7 Å. **h**, Final 3D reconstruction filtered and colored according to local resolution. **i**, Fourier shell correlation curves of showing unmasked (No mask) and masked (Corrected) FSC curves.

Next, we aimed to reconstitute in yeast a hexameric 5S RNP formed with only the human orthologous proteins in order to study its assembly independently of the complexity of human cells. For this purpose, we co-overexpressed in yeast the genes for human Syo1 (called HEATR3), Rpf2, Rrs1, uL18 and uL5 and performed split-tag affinity purification using *hs*uL5 as first bait and *hs*Rpf2 as second. Similar to the fungal complexes, this 5S RNP was a hexamer composed of *hs*Syo1, *hs*Rpf2, *hs*Rrs1, *hs*uL18, *hs*uL5 and the yeast 5S rRNA, which is structurally well conserved between *S. cerevisiae* and *Homo sapiens* (*hs*) (Fig. 1e). Next, we expressed these human factors in the otherwise lethal *rpf2*Δ knockout strain, where *hs*Rpf2 was able to complement the non-viable yeast *rpf2*Δ mutant, but with a reduced growth rate (Extended Data Fig. 3a, b). This enabled us to not only isolate the free 5S RNP hexamer with exclusively human proteins, *hs*Syo1–*hs*Rpf2–*hs*Rrs1–*hs*uL18–*hs*uL5, but also enrich for yeast pre-60S particles that bound to the co-assembly of human factors (Extended Data Fig. 3c, sucrose gradient fraction 13). Cryo-EM analysis of this unusual intermediate revealed a composite human–yeast 5S RNP without *hs*Syo1 at the central protuberance of the yeast pre-60S ribosome in a typical prerotated conformation (Extended Data Fig. 3d–i and Table 1). However, when comparing with related wild-type yeast pre-60S particles^14^, the 5S RNP with the human factors was not as rigidly incorporated as the endogenous yeast 5S RNP. The lower sequence conservation between the C-terminal extensions of yeast and human Rpf2 and Rrs1 might determine a more flexible, less optimal anchoring of the chimeric 5S RNP to the underlying yeast pre-60S core structure. This interpretation is consistent with the finding that the *rpf2*Δ knockout strain is not fully complemented by human Rpf2 (Extended Data Fig. 3a, b).

Altogether, these data suggest that the conserved 5S RNP is a hexamer (Syo1–Rpf2–Rrs1–uL18–uL5–5S rRNA), in which the inclusion of Syo1 and Rpf2–Rrs1 might render the nascent 5S RNP competent for incorporation into the pre-60S subunit.

### Cryo-EM structure of the conserved 5S RNP hexamer

To gain insight into the architecture of the conserved 5S RNP hexamer precursor, we focused on the 5S RNP isolated from *C. thermophilum* under normal growth conditions. The cryo-EM structure of this complex was solved at an average resolution of 3.5 Å (Fig. 2a, Extended Data Fig. 1d–h and Table 1), which allowed us to build a near-complete molecular model (Fig. 2b). As starting models, we extracted the yeast 5S rRNA–uL18–uL5–Rpf2–Rrs1 complex from a pre-60S cryo-EM structure (Protein Data Bank (PDB) ID 3JCT^10^) and used the crystal structure of the *C. thermophilum* Syo1–uL5 complex (PDB ID 4GMN)^15^. In addition, we solved the cryo-EM structure of the hexameric *ct*–*sc* 5S RNP chimera to a resolution of 4.1 Å (Extended Data Fig. 2 and Table 1). This preparation contained the expected hexamer, as well as dimers of same hexamer. The cryo-EM structure of the dimer at a resolution of 4.0 Å revealed an identical arrangement of the two hexamers in the dimer and in the monomer (Extended Data Fig. 2h and Table 1); however, it remains unclear if a 5S RNP dimer of hexamers exists under physiological conditions.

The overall architectures of the *C. thermophilum* hexameric 5S RNP and the related *ct–sc* chimera are highly similar; both are hexameric complexes, in which the protein factors Syo1, uL18, uL5, Rpf2 and Rrs1 are positioned around the prominent and typically structured 5S rRNA (Fig. 2a,b and Extended Data Fig. 2g). Only the Rpf2–Rrs1 C-terminal extensions are not visible at normal contour levels, suggesting that they are exposed and flexible. However, at lower contour levels, an outer blurry density with connection to the Rpf2–Rrs1 heterodimer can be discerned, which might correspond to the flexible Rpf2–Rrs1 C-terminal extensions possibly involved in pre-ribosome targeting and anchoring.

### uL18–uL5 transfer from the import complex onto the 5S RNP

The overall architecture of the 5S hexameric particle adopted to a large extent the conformation of the 5S RNP as observed in the context of the pre-60S assembly intermediate^10^. Therefore, comparing the structure of the 5S RNP hexamer with that of the previously characterized *ct*Syo1–*ct*uL5–*ct*uL18-N import complex^16^ revealed the required transition between these two states (Fig. 2c). One significant change brought about by this transition is the repositioning of the uL18 N terminus (residues 2–30), which in the import complex is bound to the inner surface of the Syo1 α-solenoid^16^ but is located elsewhere in the hexameric 5S RNP. The major domains of uL18 (central and C-terminal domains) also become tightly bound to the 5S rRNA, leading to an overall arrangement similar to that in the mature 60S subunit. This explains why, after repositioning, the uL18 N terminus does not reach into its previous Syo1 docking site and only the N-terminal tip can remain in contact with the nearby Syo1 C-terminal α-helix (residues 650–672) (Fig. 2c). Consistent with this relocation, the Syo1 internal concave surface is unoccupied in the *ct–sc* chimera 5S RNP hexamer (Extended Data Fig. 2g), but an extra density persists at this site in the *C. thermophilum* 5S RNP hexamer (Fig. 2a). Whether this density is an additional uL18-N peptide of unknown origin, or is derived from another factor (for example, part of the flexible Syo1 loop), remains unclear.

Like uL18, uL5 becomes firmly attached to the 5S rRNA. However, during its relocation from the import complex, uL5 remains tethered to Syo1 via a helical motif (residues 389–398) that is part of the flexible Syo1 acidic loop, which inserts into the β-sheeted uL5 groove^16^ (Fig. 2c). Although the local resolution in this region does not allow side-chain identification, we can clearly follow the emergence of Syo1’s acidic loop from an internal Syo1 HEATR helix (at residue P412) and its connection to the helical motif (residues 392–400) integrated within the uL5 groove (Fig. 2c). This finding suggests that the Syo1 helical motif prevents the critical uL5 groove from engaging prematurely with H84 of the 25S rRNA, or other factors, prior to pre-60S assembly.

We performed crosslinking mass spectrometry (XL-MS) of the purified *C. thermophilum* and *S. cerevisiae* hexameric 5S RNPs to complement our structural models (Fig. 1f and Source Data Excel file). Overall, this analysis revealed similar crosslink patterns between the 5S RNP factors in the two hexameric complexes, indicating a similar conserved structure. However, we noticed a few differences. As an example, in the yeast 5S RNP hexamer, uL5 exhibits a higher number of intermolecular and intramolecular self-crosslinks and inter-crosslinks with the uL18 N-terminal extension. In the thermophile complex, only a few of these uL5 crosslinks were detected, which is well supported by the *C. thermophilum* hexameric 5S RNP cryo-EM structure. Although these observations could be explained by preparation-specific variations, they could also suggest a subtle variation in the assembly states or differences in regulation of 5S RNP incorporation into pre-ribosomes.

A final structural rearrangement during 5S RNP construction appears to involve Syo1’s flexible N terminus including the PY-NLS (residues 1–15), which is not part of the *ct*Syo1–*ct*uL5–*ct*uL18-N crystal structure^16^ but is most likely located in the region of the tip of the 5S rRNA (loop D in helix IV) according to our cryo-EM structure (Fig. 2c). Masking of this site by its binding to 5S rRNA may hinder rebinding of the Syo1 NLS to its import receptor Kap104 in the nucleus, suggesting a mechanism by which Syo1 changes from an import factor to an assembly factor.

### Role of Syo1 in the 5S RNP hexamer

**Extended Data Fig. 4:**
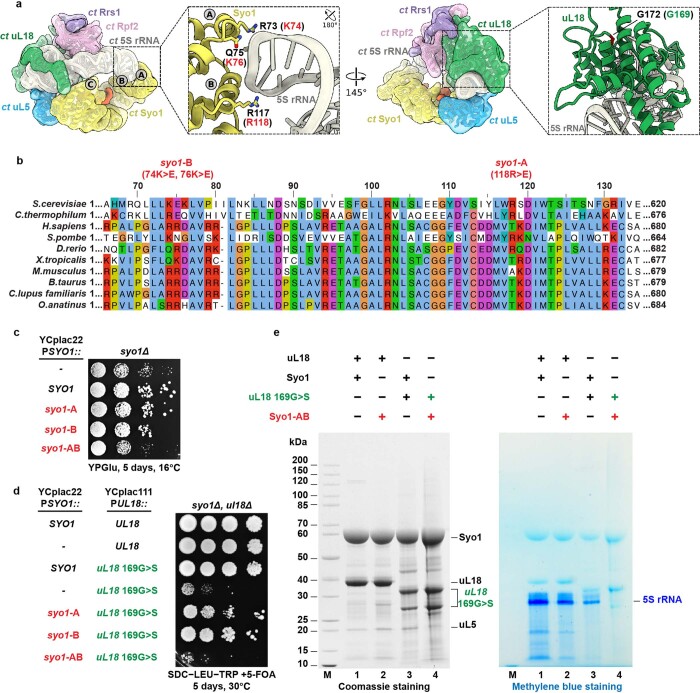
Structure-based mutations in Syo1 mapping at the contact site to 5S rRNA helix IV. **a**, Overview and zoomed-in view of the ct 5S RNP cryo-EM structure showing contact of Syo1 N-terminal end to the 5S rRNA tip in the region of helix IV. Sites A and B correspond to regions in the Syo1 N-terminal domain with direct contact to the 5S rRNA. The residues of sites A and B for ctSyo1 are labeled in black and the corresponding residues in yeast Syo1 in red. Site C corresponds to a region in C-terminal domain of Syo1 that is also in close proximity to the 5S rRNA. Highlighted is also residue G169 of S. cerevisiae uL18 (G172 in C. thermophilum), which upon mutation yields a slow-growing yeast mutant (see also panel D). **b**, Multiple sequence alignment of the Syo1 N-terminal region, indicating the positively charged amino acids at the 5S rRNA contact sites: K74 and K76 (termed syo1-B) and R118 (termed syo1-A). These sites were mutated to glutamic acids. **c**, Dot–spot growth analysis of the syo1∆ strain transformed with plasmid-borne SYO1, syo1-B (74K>E, 76K>E), syo1-A (118R>E), and the combination syo1-AB (74K>E, 76K>E, 118R>E). Growth analyses have been performed twice with a similar outcome. **d**, Synthetic lethality relationship between syo1-AB and uL18 169G>S mutant alleles. The yeast syo1∆ uL18∆ double-disruption strain (carrying wild type uL18 on pRS316-URA3) was transformed with plasmids carrying either uL18 or uL18 169G>S in combination with plasmids carrying either empty (-), SYO1 or the indicated syo1 mutant alleles. This genetic experiment has been repeated twice with a similar outcome. **e**, Split-tag affinity purification of overexpressed Syo1 or Syo1-AB mutant (both ProtA-TEV-tagged) in combination with uL18 or uL18 169G>S (both FLAG-tagged). The final eluate was analyzed by SDS-PAGE and Coomassie staining to reveal Syo1 and uL18 (left panel), and methylene blue staining to visualize the 5S rRNA (right panel). The uL18 169G>S mutant protein is partially degraded, yielding two smaller breakdown products verified by mass spectrometry. This co-immunoprecipitation assay has been performed twice with a similar outcome. Source data

The cryo-EM structure of the 5S RNP hexamer shows direct contacts between Syo1 and the 5S rRNA: two contacts ‘A’ and ‘B’ in the Syo1 N terminus that touch the 5S rRNA at loop D in helix IV, and another contact ‘C’ between a Syo1 C-terminal helix (residues 650–671) and the 5S rRNA middle region (Extended Data Fig. 4a). To verify the functionality of these contacts, we mutated the corresponding sites in yeast *syo1*-A (R118E) and *syo1*-B (K74E, K76E) separately (Extended Data Fig. 4b), but did not observe a growth defect (Extended Data Fig. 4c). However, when the mutations were combined (*syo1*-AB), a complete loss of Syo1’s in vivo function was observed, indicated by a slow growth phenotype similar to the *syo1*Δ null strain (Extended Data Fig. 4c). Moreover, a synthetic lethal phenotype was observed when the *syo1*-AB allele was combined with the otherwise viable *uL18* G169S, which was previously identified as a viable mutant that destabilizes the 5S RNP if Syo1 function is disrupted^15^ (Extended Data Fig. 4d).

We further biochemically investigated the genetic relationship between *syo1*-AB and *uL18* G169S. This revealed strongly reduced 5S rRNA co-precipitation if the synergistic Syo1 K74E/K76E/R118E (*syo1-*AB) mutant was affinity purified in combination with uL18 G169S (Extended Data Fig. 4e). Thus, residues in the Syo1 α-solenoid that mediate 5S rRNA binding are crucial for Syo1’s in vivo function, likely by recruiting 5S rRNA for 5S RNP biogenesis and/or by facilitating assembly into the pre-ribosomes.

### Assembly of the hexameric 5S RNP into pre-60S particles

**Fig. 3:**
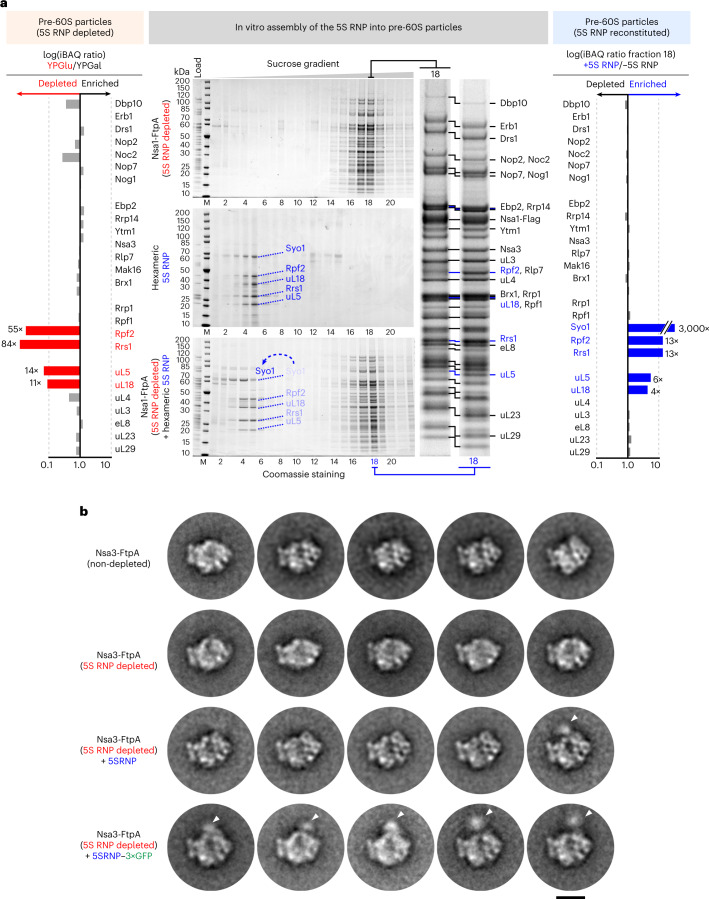
In vitro assembly of the yeast hexameric 5S RNP into early nucleolar pre-60S particles depleted of endogenous 5S RNP. **a**, Left: SQ-MS analysis of the pre-60S particles depleted for the 5S RNP and used for the in vitro assembly (affinity purified via Nsa1-FtpA, derived from the *GAL-HA-RPF2* yeast strain) compared with the same 5S RNP-non-depleted particles (Extended Data Fig. 5 and Source Data Excel file). Middle: in vitro assembly of the reconstituted yeast hexameric 5S RNP into the pre-60S particles depleted of 5S RNP. Samples were subjected to sucrose gradient centrifugation, fractioned and analyzed by SDS–PAGE. Labeled bands were identified by mass spectrometry. Right: SQ-MS analysis of pre-60S particles after binding of the 5S RNP compared with the non-reconstituted particles (fraction 18 of the sucrose gradients) (Source Data Excel file). Because Syo1 is absent in the Nsa1-FtpA sample, small amounts of Syo1 detected in the reconstituted sample (+5S RNP) may explain the high enrichment factor, which accordingly should be interpreted with caution. The iBAQ values were normalized to Erb1 in both SQ-MS analyses (left and right). The in vitro binding assay was repeated at least ten times with consistent results. **b**, Negative-stain EM of the pre-60S particles before and after 5S RNP depletion and after 5S RNP reconstitution. The assay was performed with 5S RNP-depleted pre-60S particles (affinity purified via Nsa3-FtpA from the *GAL-HA-RPF2* strain) and 5S RNP containing uL18 untagged or tagged with three GFP moieties (3×GFP). The portions of the sucrose gradient with high molecular weight were analyzed by negative-stain EM, from which 2D class averages showed pre-60S alterations in the 5S RNP region. Specifically, pre-60S particles reconstituted with the 5S RNP–3×GFP exhibited an extra density (indicated by white arrows) that corresponds to the central protuberance of the pre-60S. Scale bar, 20 nm. For the entire 2D classes dataset, see Supplementary Information.

**Extended Data Fig. 5:**
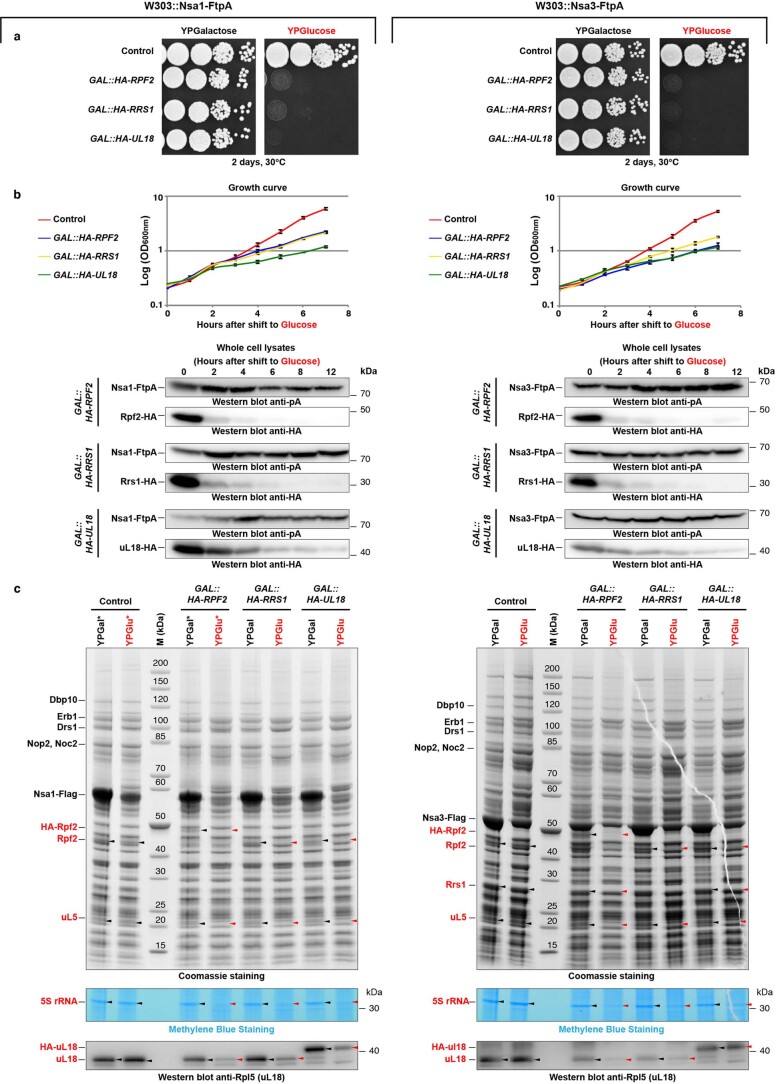
In vivo depletion of the yeast 5S RNP from pre-60S particles by GAL promoter-driven repression of either GAL::HA-RPF2, GAL::HA-RRS1 or GAL::HA-uL18. **a**, **b**, Yeast strains (W303) carrying genomically integrated FLAG-TEV-ProtA tag fused at the C-terminus of either NSA1 (left) or NSA3 (right), each under control of the endogenous promoter. Dot–spot (a) and liquid culture (b) growth analysis of the indicated yeast strains on galactose (inducing) and glucose (non-inducing) containing medium. Samples were collected at the indicated hours and whole lysates were analyzed by western blot (lower panel) to follow the depletion of the indicated gene constructs. The growth curves show the mean ± STD of 3 replicas per culture. **c**, SDS-PAGE analysis of pre-60S particles isolated via tandem affinity purification based FtpA-tagged bait constructs (left: Nsa1-FtpA; right, Nsa3-FtpA) of the corresponding yeast strains, grown after 6 hours in either glucose or galactose containing medium. Black arrows point to the 5S RNP factors, and red arrows to the verified depletion (analyzed by mass spectrometry) of these factors. Labeled bands were identified by mass spectrometry. Staining with methylene blue revealed the 5S rRNA, and uL18 was detected by Western blot. Total YPGal and YPGlu eluate samples marked with asterisks were analyzed by SQ-MS. (Source Dataset 2; see also Fig. 3a, left panel). These depletion experiments have been done at least twice for all the groups with consistent results, and the Rpf2-depletion was further used as routine for the production of pre-60S particles depleted of 5S RNP. Source data

To test whether the hexameric 5S RNP is the precursor that assembles into the pre-ribosome, we aimed to reconstitute this process in vitro. We generated early pre-60S particles devoid of the entire 5S RNP, by repression of any of the 5S RNP components (Extended Data Fig. 5)^6^. Accordingly, we placed *RPF2* under the control of the regulatable *GAL* promotor in yeast (*GAL*::*HA-RPF2*) and affinity purified the early nucleolar pre-60S particles via the pre-60S factor Nsa1 (Extended Data Fig. 5). As anticipated, these pre-ribosomes were depleted of Rpf2, Rrs1 and the minimal 5S RNP core (uL18–uL5–5S rRNA), but other pre-60S factors were not or only marginally affected (Fig. 3a, left and Extended Data Fig. 5c). Next, we incubated these 5S RNP-depleted pre-60S particles with purified hexameric 5S RNP, and analyzed on a sucrose gradient the binding of the reconstituted ribosomal complex (Fig. 3a, middle). Whereas in the mock controls the free 5S RNP or pre-60S ribosomes migrated to their expected positions on the gradient, upon mixing, the 5S RNP and pre-60S particles co-migrated, showing reconstitution of the 5S RNP into pre-ribosomes (Fig. 3a, middle and right, fraction 18).

**Extended Data Fig. 6:**
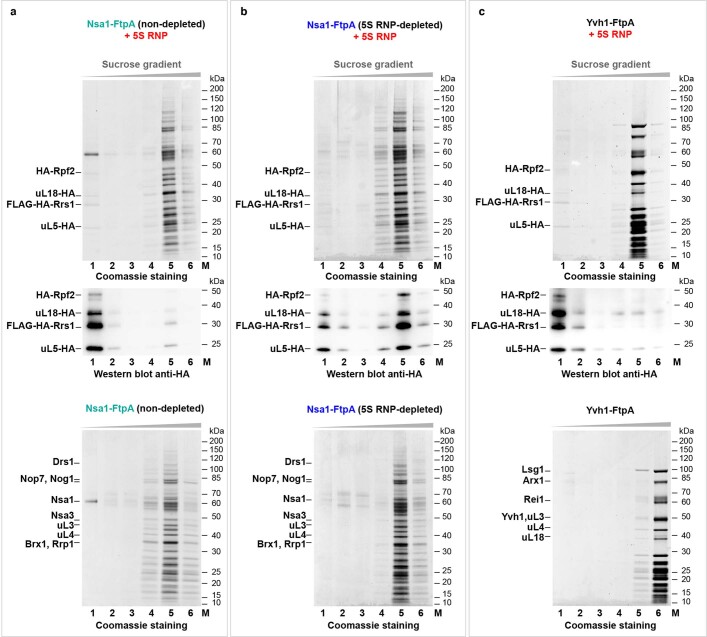
Yeast 5S RNP in vitro binding reconstitution into pre-60S particles using epitope-labeled subunits. **a**–**c**, The yeast hexameric 5S RNP was incubated with Nsa1-FtpA pre-60S particles, either non-depleted (a) or 5S RNP-depleted via GAL::HA-RPF2 (b), and as a further control, with Yvh1-FtpA-derived pre-60S particles (c). After sucrose gradient centrifugation of the assay mixtures, all fractions (fractions 1–6) were analyzed by SDS-PAGE (upper panel), and western blotting using the anti-HA antibody (middle panel). The bands corresponding to HA-Rpf2, uL18-HA, FLAG-HA-Rrs1, and uL5-HA are indicated. The different Nsa1-FtpA and Yvh1-FtpA pre-60S particles without addition of 5S RNP were also analyzed by sucrose gradient centrifugation (lower panels). M: Molecular weight marker. This in vitro binding assay has been done at least twice with a similar outcome.

In addition, we set up a modified reconstitution assay, in which binding could be directly monitored by western blotting, using a 5S RNP hexamer assembled with epitope HA-tagged subunits (Extended Data Fig. 6). This modified assay also demonstrated significant association of the 5S RNP factors to the Rpf2-depleted pre-60S particles (Extended Data Fig. 6b). However, minimal 5S RNP binding was observed to either non-depleted Nsa1 particles (Extended Data Fig. 6a) or late cytoplasmic Yvh1-derived pre-60S particles (Extended Data Fig. 6c), which already carry the mature and tightly integrated 5S RNP at the central protuberance^26^. Together, these data demonstrate that the 5S RNP binding to a distinct population of pre-60S ribosomes lacking the 5S RNP can be reconstituted in vitro with high specificity.

Prompted by these findings, we aimed to visualize the reconstituted 5S RNP on these pre-60S particles. Single-particle cryo-EM could not be used to achieve this, because the 5S RNP is not visible in averages of nucleolar pre-60S particles owing to its flexible association at the immature central protuberance^27,28^. To make the resulting reconstituted 5S RNP hexamer more distinguishable on the pre-60S particles by negative-stain EM, we also tagged uL18 with three concatenated green fluorescent protein (GFP) moieties (uL18–3×GFP). Following this strategy, a number of the two-dimensional (2D) classes of the negative stained pre-60S particles reconstituted with the 5S RNP–3×GFP exhibited an extra density at a discrete region that corresponds to the central protuberance of the pre-60S (Fig. 3b and Supplementary Information). In rarer cases, such an extra density was also seen for the reconstituted 5S RNP not carrying the 3×GFP, but never observed in the case of the pre-60S particles only depleted for the 5S RNP (Fig. 3b and Supplementary Information). We interpret this finding as evidence that the 5S RNP is recruited to a specific site on pre-60S, which also corresponds to the position in nascent 60S subunits to which the 5S RNP is normally bound.

**Fig. 4:**
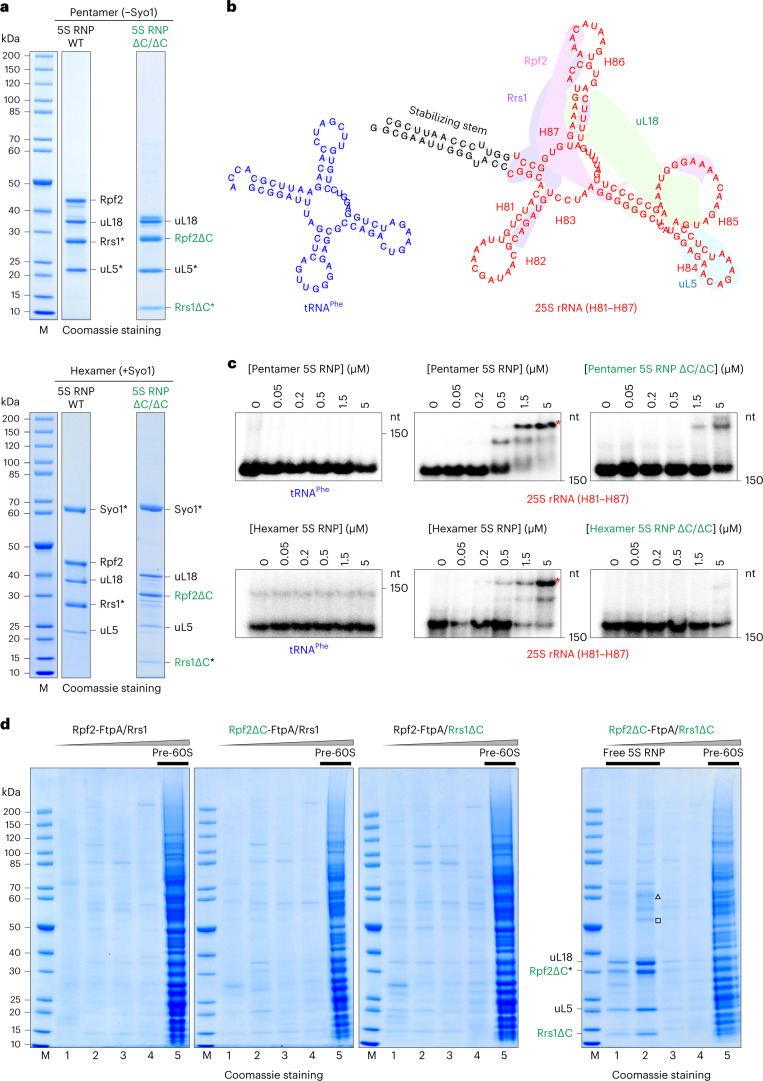
Rpf2–Rrs1 C-terminal extensions are required for 5S RNP assembly into pre-60S ribosomes. **a**, SDS–PAGE analysis of the yeast 5S RNP complexes, Syo1 lacking (top) or Syo1 containing (bottom), with either wild-type (WT) or truncated (ΔC/ΔC) Rpf2/Rrs1 factors, used for the EMSA. Asterisks indicate bait proteins used for the split-tag affinity purification. The purifications of these 5S RNP complexes were done more than three times with consistent results. **b**, Folding prediction, calculated by RNAfold^46^, of the yeast 25S rRNA fragment H81–H87 (red, right) and tRNA^Phe^ (blue, left) used for the EMSA with the 5S RNP complexes. The colored areas illustrate the contacts of the 5S RNP proteins to the 25S rRNA helixes (labeled from H81 to H87) after binding to pre-60S ribosome (PDB ID 3JCT). **c**, EMSA radiographs showing the specific band shift (asterisks) of the radiolabeled 25S rRNA fragment upon binding to increasing amounts of the indicated 5S RNP complexes. nt, nucleotides. All of the EMSA assays were done twice with a similar outcome. **d**, SDS–PAGE analysis of sucrose gradient fractions from in vivo 5S RNP assembly into pre-60S ribosomes in yeast cells. 5S RNP incorporation into pre-60S particles was monitored upon affinity purification using Rpf2 as bait, from yeast wild-type, *rpf2∆C* and *rrs1∆C* single mutants, and *rpf2∆C rrs1∆C* double mutants. The typical protein pattern of pre-60S ribosomes is visible in the fractions with high molecular weight (lane 5), whereas the free 5S RNP (unbound) is visible in the fractions with low molecular weight (lanes 1 and 2). The bands corresponding to the 5S RNP factors were identified by mass spectrometry and are labeled. Co-precipitation of Fpr3 (triangle) and Fpr4 (square) with the free 5S RNP was also detected. This in vivo binding experiment was done at least twice with a similar outcome. Source data

To elaborate on the idea that the 25S rRNA of the central protuberance (H81–H87) serves as an initial docking site for the 5S RNP^27,28^, we used an in vitro RNA band-shift assay. For this electrophoretic mobility shift assay (EMSA), we in vitro transcribed H81–H87 and connected the respective 5′ and 3′ ends with a stable GC stem and incubated this RNA with reconstituted 5S RNP preparations (Fig. 4a, b). As a negative control, we in vitro transcribed an also highly structured transfer RNA (tRNA) of similar size (Fig. 4b). We observed a robust shift of the H81–H87 RNA band after incubation with increasing amounts of either the isolated yeast 5S RNP hexamer (containing Syo1) or pentamer (lacking Syo1), whereas the tRNA was not shifted even at the highest concentrations of 5S RNPs (Fig. 4c). Notably, for the isolated 5S RNP assembled with Rpf2–Rrs1 lacking their C-terminal extensions (5S RNP ∆C/∆C; either pentamer or hexamer), the shift of the H81–H87 fragment band was largely reduced (Fig. 4c), which is consistent with these extensions having a role in the recruitment of the 5S RNP to pre-60S particles.

To further examine the in vivo role of the Rpf2–Rrs1 C-terminal extensions, we affinity purified pre-60S ribosomes via Rpf2-FtpA, using yeast expressing Rpf2∆C, Rrs1∆C or a combination of both mutants (Fig. 4d). The 5S RNP in vivo was not efficiently incorporated into pre-60S particles if both C-terminal extensions from Rpf2 and Rrs1 were removed and accumulated instead as a free complex running in the fractions with lower molecular weight after sucrose gradient centrifugation (Fig. 4d). Altogether, these data suggest that the C-terminal extensions of Rpf2–Rrs1 help target and tether the 5S RNP during assembly into nucleolar pre-60S particles.

### Structure of human 5S RNP bound to ubiquitin ligase Mdm2

**Fig. 5:**
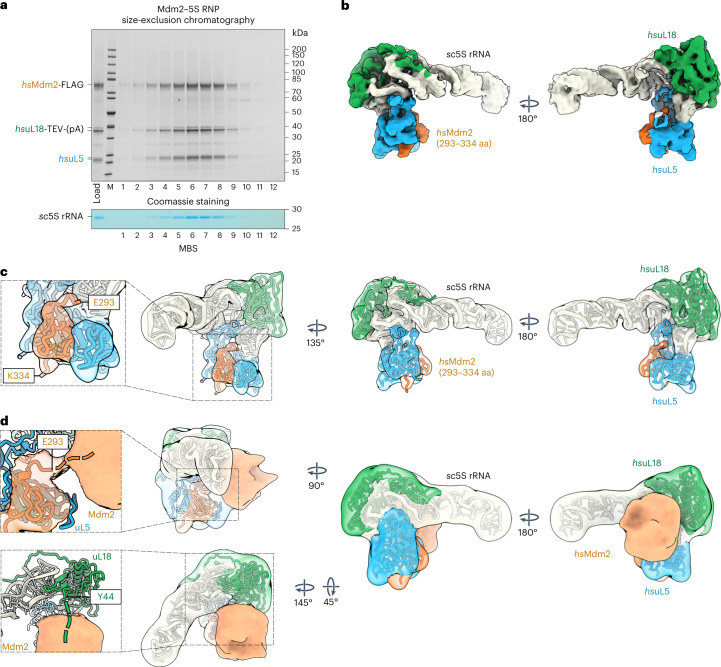
Reconstitution and cryo-EM structure of the Mdm2–5S RNP complex. **a**, Split-tag affinity purification of the reconstituted Mdm2–5S RNP complex (*hs*Mdm2*–hs*uL18–*hs*uL5–*sc*5S rRNA) using *hs*uL18-TEV-ProtA as first bait and *hs*Mdm2-FLAG as second bait, followed by size-exclusion chromatography. The final eluate (Load) and fractions 1–12 from the gel-filtration column were analyzed by SDS–PAGE. Labeled bands were identified by mass spectrometry. The gel was also stained with MBS to reveal the 5S rRNA. The purification of the Mdm2–5S RNP complex was performed more than five times with similar outcomes. **b**,**c**, Cryo-EM map (**b**) and fitted model (**c**) of the Mdm2–5S RNP complex (PDB IDs 4XXB for Mdm2, 6ZM7 for *hs*uL5 and *hs*uL18, and 3JCT for *sc*5S rRNA). aa, amino acids. The components of the complex are shown in different colors and labeled. **d**, Model and Gaussian filtered map of the Mdm2–5S RNP complex refined without mask and at lower contour levels revealing additional Mdm2 electron density. The appearing flexible density (unresolved parts of Mdm2) contacts the N-terminal residue E293 of the Mdm2 zinc finger domain, as well as the uL18 N-terminal residue Y44 (expansions, left). The connections of the Mdm2 zinc finger and the unresolved N terminus of uL18 are indicated with dashed lines. Source data

Based on our structural and functional insights into the conserved 5S RNP, we proceeded to test whether we could reconstitute and isolate a free 5S RNP that instead carries the E3 ubiquitin ligase Mdm2, which in human cells is known to regulate p53 levels under different stress conditions^29–32^ (including disturbed ribosome assembly). So far, only one crystal structure of a complex between uL5 and a short motif from the Mdm2 middle domain has been reported^33^. An Mdm2-bound 5S RNP has remained elusive both structurally and biochemically, despite many lines of evidence suggesting its existence and importance^21^. To reconstitute this putative intermediate, we co-expressed *hs*Mdm2 (FLAG-tagged), *hs*uL5 (untagged) and *hs*uL18 (TEV-ProtA-tagged) in yeast, which together readily assembled into a Mdm2–5S RNP complex that was subsequently isolated by split-tag affinity purification and further fractionated by size-exclusion chromatography (Fig. 5a). We observed strict co-elution of the four components *hs*Mdm2, *hs*uL18, *hs*uL5 and *sc*5S rRNA, thus suggesting that the otherwise elusive Mdm2–5S RNP complex is a biochemically distinct and stable entity.

**Extended Data Fig. 7:**
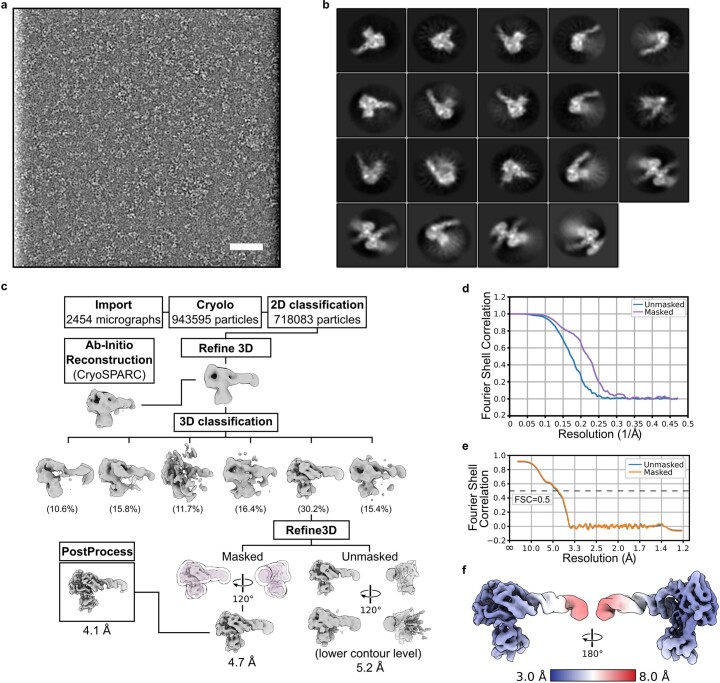
Cryo-EM analysis of the Mdm2-5S RNP complex. **a**,**b**, Representative micrograph of the Mdm2-5S RNP assembly low-pass filtered to 20 Å, displayed with inverted contrast (out of 2454 micrographs used for processing) (a) and selected 2D classification averages (b). Scale bar: 50 nm. **c**, Cryo-EM processing scheme of the Mdm2-5S RNP. The overall resolution of the final 3D reconstruction was 4.1 Å. **d**, FSC curves of the final 3D reconstructions showing masked and unmasked FSC curves. **e**, Model-to-map FSC curves. **f**, Final 3D reconstruction filtered and colored according to local resolution.

We further solved the structure of the Mdm2–5S RNP complex by cryo-EM at an average resolution of 4.1 Å (Fig. 5b–d, Extended Data Fig. 7 and Table 1), which was sufficient to reveal how the Mdm2 zinc finger middle domain (residues 293–334) is integrated with the β-sheeted concave surface of *hs*uL5, as observed in the crystal structure (Fig. 5c)^33^. Furthermore, our cryo-EM density map shows how *hs*uL5 and *hs*uL18 remain attached to their cognate sites on the 5S rRNA while mediating contact with Mdm2 (Fig. 5b). Notably, at lower contour levels or when filtered to low resolution, a globular density emerged that is connected to the Mdm2 zinc finger middle domain and located at the 5S rRNA middle stem, adjacent to uL18 (Fig. 5d). Interestingly, a number cancer-associated mutations in the ribosomal protein uL18 dysregulate the MDM2/p53-mediated ribosome biogenesis checkpoint^34^. We attribute this additional density in our Mdm2–5S RNP structure to the remainder of Mdm2, that is, the Mdm2 N-terminal (that is, the p53 binding domain), acidic and C-terminal RING finger (ubiquitin ligase) domains. Notably, this flexible Mdm2 density partly overlaps with the Rpf2–Rrs1-binding region, thus suggesting a competition between Mdm2 and the Rpf2–Rrs1 dimer for joining the 5S RNP.

**Fig. 6:**
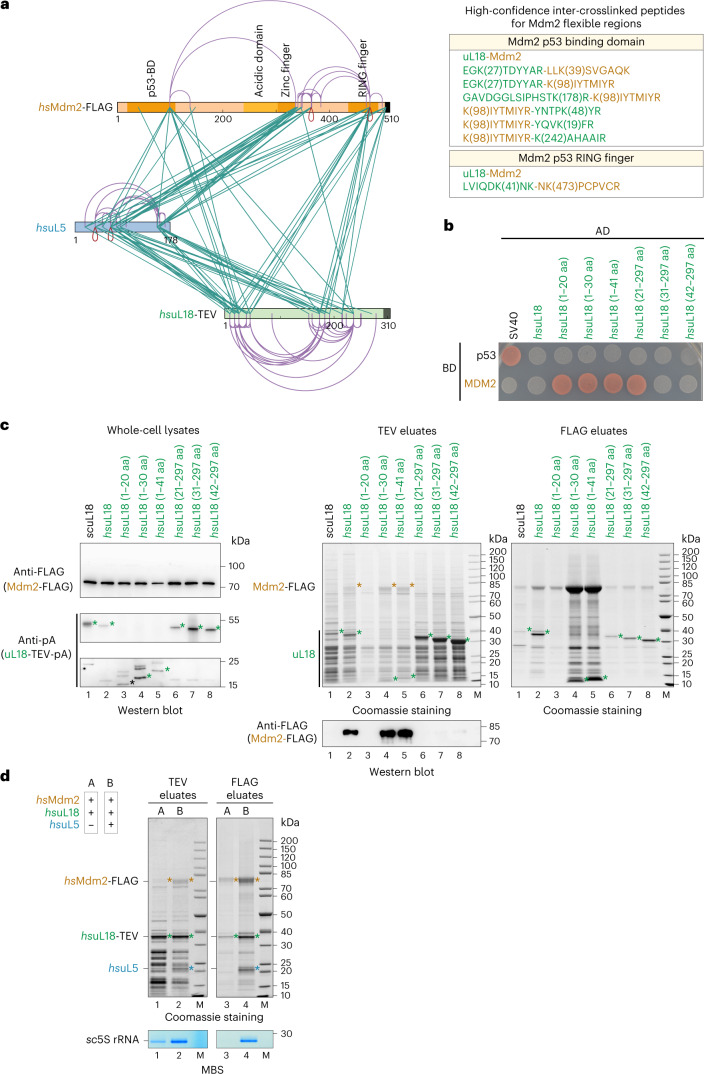
The human uL18 N-terminal sequence specifically binds to the Mdm2 N domain. **a**, Left: XL-MS of the purified Mdm2–5S RNP complex, using DSS-H12. All inter-crosslinks and self-crosslinks are depicted. The xiNET tool^45^ was used for visualization of the crosslinks and the primary structure of the proteins. The domain organization of Mdm2 is also displayed. Right: manually curated list of the high-confidence inter-crosslinked peptides for the flexible regions of Mdm2 unresolved by cryo-EM. **b**, Yeast two-hybrid interaction between the indicated *hs*uL18 constructs and *hs*Mdm2. AD, activation domain; BD, binding domain. The yeast two-hybrid assay was performed twice with a similar outcome. **c**, Sequence-specific binding of *hs*uL18 to *hs*Mdm2, analyzed by co-expression and pull-down assays in yeast cells. *GAL*-induced co-expression of *hs*uL18 N-terminal constructs (fused at the C terminus to TEV-ProtA) and *hs*Mdm2-FLAG, followed by IgG Sepharose chromatography and TEV cleavage (TEV eluates). Total lysates (left) were analyzed by western blotting for *hs*uL18-TEV-ProtA and Mdm2-FLAG using anti-ProtA and anti-FLAG antibodies, respectively. The TEV eluates were further affinity purified on FLAG beads to enrich for Mdm2-FLAG. Both TEV (middle) and FLAG (right) elutes were analyzed by SDS–PAGE. Mdm2 and uL18 bands are indicated by orange and green asterisks, respectively. The FLAG-labeled Mdm2 bands in lanes 2, 4 and 5 (orange asterisks) of the Coomassie-stained gel (TEV eluates) were also verified by mass spectrometry. This co-immunoprecipitation assay was performed twice with similar outcomes. **d**, Cooperative binding of *hs*uL5 and *hs*uL18 to Mdm2, analyzed by co-expression and pull-down assays in yeast cells. Sample A corresponds to the co-expression of *hs*uL18 and *hs*Mdm2, whereas in sample B, *hs*uL5 was added to the in vivo co-expression system. Tandem affinity purifications from the cell lysates were performed by pulling down *hs*uL18-TEV-ProtA (TEV eluates) in the first step and *hs*Mdm2-FLAG (FLAG eluates) in the second step. Eluates were analyzed by SDS–PAGE and Coomassie staining or methylene blue staining. Mdm2, uL18 and uL5 bands are indicated by orange, green and blue asterisks, respectively. This co-immunoprecipitation assay was performed twice with similar outcomes. Source data

To investigate how the more flexible part of Mdm2 establishes its contact points to the 5S RNP and whether the relocated uL18 N terminus (see Fig. 5d) is directly involved in this tethering, we again performed complementing XL-MS (Fig. 6a and Source Data Excel file). In general, the identified crosslinks strongly support our structural model of the Mdm2–5S RNP complex, with contacts of the various Mdm2 domains to both uL18 and uL5. Notably, several of the top-scoring crosslinks were found between the Mdm2 N terminus and the uL18 N-terminal extension (Fig. 6a, right), which could point to a direct contact. Therefore, we determined whether Mdm2 can directly interact with uL18 by performing two-hybrid assays (Fig. 6b) and affinity purifications (Fig. 6c). The uL18 fragment residues 1–20 are sufficient to bind to Mdm2 by two-hybrid assay, but for a stable biochemical purification, uL18 residues 1–30 are required. Conversely, uL18 residues 31–297 no longer interact with Mdm2 by two-hybrid assay and biochemical reconstitution, whereas the slightly longer uL18 (21–297) construct was still active in the two-hybrid assay but not in the biochemical assay. These differences between two-hybrid assay and co-immunoprecipitation for some borderline constructs might be due to the harsher biochemical purification conditions, but overall both assays revealed that the uL18 N-terminal extension (1–30) efficiently binds to Mdm2, whereas the uL18 core (residues 31–297) does not. Based on these findings, we wondered whether uL18–Mdm2 binding is cooperatively enhanced by the presence of uL5, which might support a potential cooperative effect suggested in previous studies^35,36^. For this purpose, we performed an in vivo binding assay based on heterologous co-expression in yeast of human uL18 and Mdm2, with or without human uL5 (Fig. 6d). Evidently, *hs*uL18 and *hs*Mdm2 interacted in the absence of *hs*uL5 only inefficiently, but when *hs*uL5 was co-expressed, Mdm2–5S RNP (uL18, uL5 and 5S rRNA) complex formation was significantly increased (Fig. 6d). Thus, Mdm2 exhibits more than one contact point within the 5S RNP: one to uL5 involving the middle zinc finger domain and another one to the uL18 N-terminal extension (Fig. 5d). Both of these contacts overlap with the Syo1 binding regions, which also explains structurally why binding of Mdm2 or Syo1 to the 5S RNP is mutually exclusive. Since the uL18 N terminus binds to Syo1 in the Syo1-import complex^15,16^ and the 5S RNP hexamer, as well as to the 5S rRNA within pre-ribosomes^10^, this uL18 N-terminal extension is versatile and likely able to perform a regulatory role in 5S RNP assembly (see 'Discussion').

## Discussion

**Fig. 7:**
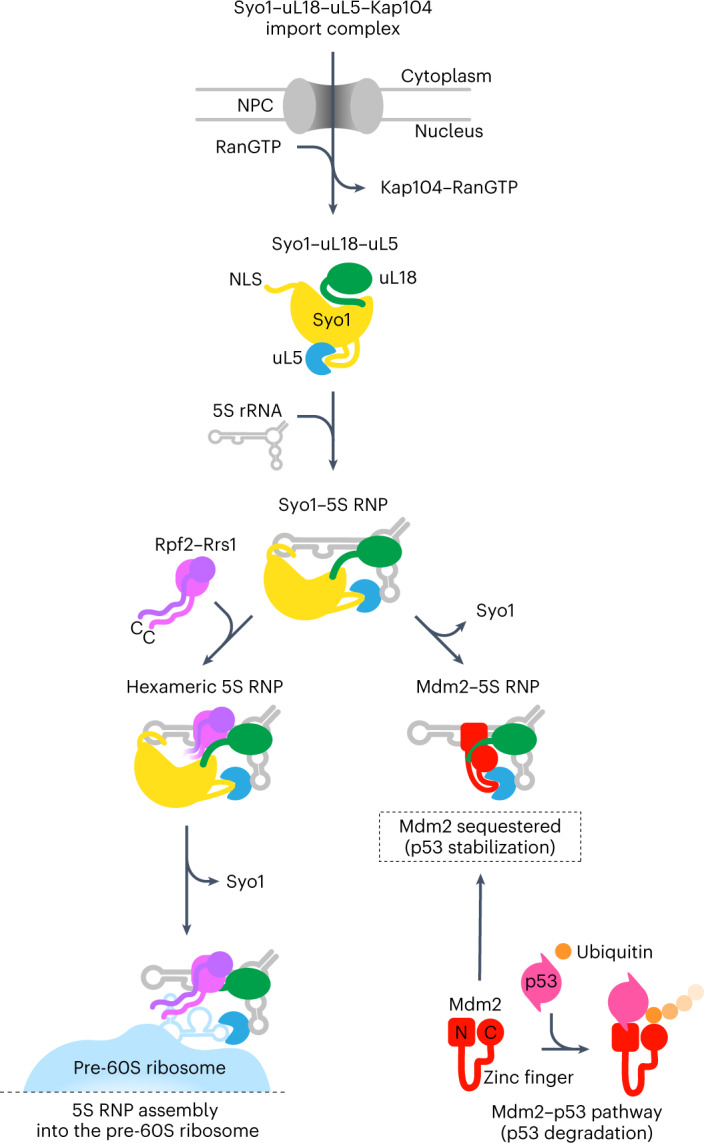
Model of the formation of 5S RNP that assembles into nascent 60S subunits or signals the MDM2–p53 pathway. After Syo1-mediated nuclear import of uL5–uL18, the 5S RNP assembles upon binding to the nascent 5S rRNA. Under normal circumstances (for example, cell proliferation), the 5S RNP is targeted to the ribosome biogenesis pathway by the binding of Rpf2–Rrs1, thereby forming the conserved hexameric 5S RNP that eventually integrates into pre-60S particles. Under nucleolar stress (for example, impaired ribosome biogenesis), the 5S RNP instead accumulates in a free pool that can sequester the ubiquitin ligase Mdm2, which in consequence stabilizes p53 levels that can eventually cause cell cycle arrest and apoptosis (see Discussion). NPC, nuclear pore complex.

In this study, we reveal the cryo-EM structure of the conserved 5S RNP hexamer and how this intermediate assembles into the pre-60S ribosome. In addition, we uncover the structure of another long-sought 5S RNP intermediate carrying the human ubiquitin ligase Mdm2, demonstrating how this p53-modifying enzyme becomes physically tethered to the free pool of 5S RNP that accumulates if ribosome biogenesis is impaired. Based on these findings, we present a structure-based model of how the assembly-competent 5S RNP is formed in consecutive steps and how it can sequester Mdm2 from the Mdm2–p53 pathway under conditions of nucleolar stress (Fig. 7).

The ribosomal proteins uL18 and uL5 are initially recruited to their common import adaptor Syo1/HEATR3 in the cytoplasm^15^. After Kap104-mediated nuclear transport and RanGTP-dependent release from the import receptor, the newly synthesized 5S rRNA joins the import complex by binding to uL18 and uL5, while Syo1 remains part of the nascent 5S RNP. The insertion of a short helix from the flexible Syo1 acidic loop into the uL5 groove guarantees that neither H84 of the 25S rRNA^37^ nor the zinc finger of Mdm2^33^ can prematurely bind to this promiscuous uL5 binding site. According to our model, the Rpf2–Rrs1 heterodimer is recruited subsequently, which completes the construction of the assembly-competent hexameric 5S RNP. This precursor complex can then be targeted and incorporated into the pre-60S particles, supported by the flexible Rpf2–Rrs1 C-terminal extensions, which act like tethers. Finally, insertion of H84 of the central protuberance 25S rRNA into the uL5 groove might trigger Syo1 release and complete pre-60S integration of the 5S RNP, albeit in a prerotated conformation^27,28^. Thus, in this cascade, Syo1 initially acts principally as an import adaptor for uL18–uL5, but after 5S RNP assembly, it continues to perform a chaperone role not only to shield the uL5 groove from unwanted interactions, but also to stabilize the 5S rRNA prior to incorporation into the pre-ribosome.

Another key finding of our study relates to the 5S RNP reconstituted with the human ubiquitin ligase Mdm2, which has a profound effect on the MDM2–p53 signaling pathway. We observed that Mdm2 interacts with the 5S RNP at two distinct sites, one involving the Mdm2 zinc finger domain that binds to uL5^33^ and another that engages other parts of Mdm2 and the uL18 N-terminal extension. The position of Mdm2 caught between uL5 and uL18, bridged by the 5S rRNA, could explain why the inhibition of Mdm2 requires a fully assembled 5S RNP as observed before^4,35,36^. It is possible that the same hydrophobic groove of the Mdm2 N domain, which binds a peptide from the intrinsically disordered p53 N-terminal domain, can also be occupied by the uL18-N peptide. Alternatively, other parts of Mdm2 such as the acidic domain or the C domain may bind the uL18-N extension. In addition, the Mdm2 position in complex with the 5S RNP overlaps partially with the position of Rpf2–Rrs1. In view of these contacts, Syo1/HEATR3 and Rpf2–Rrs1 might be part of this competitive interaction network—the first one, Syo1, due to its ability to bind the uL18 N-terminal extension and the uL5 groove in both the import complex^15,16^ and the hexameric 5S RNP, and the second one, Rpf2–Rrs1, which may also hinder Mdm2 positioning but instead channels the 5S RNP complex toward the ribosome biogenesis pathway. Therefore, under normal physiological conditions, the hexameric 5S RNP complex formation might prevent the 5S RNP from engaging with Mdm2 to avoid triggering p53 activation. However, under conditions of increasing the pool of free 5S RNP (for example, nucleolar stress), Syo1 and Rpf2–Rrs1 might be present in insufficient amounts or otherwise compromised to effectively compete for Mdm2 binding.

In conclusion, our biochemical and structural insights into different 5S RNP assembly intermediates allowed us to decipher important mechanistic steps of ribosome assembly and their connection with the Mdm2–p53 signaling pathway. This knowledge could have implications for the design of new drugs for specific cancers or other diseases such as human ribosomopathies^4,18,21,38–44^.

## Methods

### Materials availability

Strains and plasmids generated in this study are listed in Supplementary Table 1 and are available from the corresponding authors upon request. Growth and culture conditions for the respective experiments are described below. The materials, reagents and softwares used in this study are also listed in Supplementary Table 1.

### Isolation of 5S RNP complexes from *C. thermophilum*

Genomic tagging of 5S RNP-related factors (Rpf2, Rrs1 and Syo1) in *C. thermophilum* was performed as previously described^47^. Wild-type *C. thermophilum* was grown overnight in 100 ml of liquid medium (15 g of dextrin (potato), 5 g of tryptone, 3 g of sucrose, 1 g of peptone, 1 g of yeast extract, 0.5 g of NaCl, 0.65 g of K_2_HPO_4_∙3H_2_O, 0.5 g of MgSO_4_∙7H_2_O and 0.01 g of Fe(III)sulfate-hydrate, per liter, pH adjusted to 7.0). The grown mycelium was washed twice with protoplast buffer (0.8 M sorbitol, 0.013 M Na_2_HPO_4_, 0.045 M KH_2_PO_4_, pH 6.5) and incubated at 30 °C for 4 h in 40 ml of digestion solution (30 mg ml^−1^ lysing enzymes from *Trichoderma harzianum* (Sigma–Aldrich, L1412) and 10 mg of BSA fraction V in protoplast buffer). Protoplasts were collected by filtering through a funnel (pore size, 1 µm), followed by centrifugation (2,400 rpm, 4 °C, 8 min) with two washing steps in protoplast buffer and one in STC buffer (0.8 M sorbitol, 80 mM CaCl_2_, 10 mM Tris-HCl, pH 7.5). The pellet containing the protoplasts was resuspended in a small volume of STC buffer and for each transformation reaction, 200 μl were mixed with 2.5 μl of heparin (10 mg ml^−1^), 2 μl of spermidine trihydrochloride (50 mM), 1 μl of aurintricarboxylic acid (0.4 M) as nuclease inhibitor, 40 μl of STC/PEG solution (40% PEG 6000 (w/v) in STC buffer) and 5–10 μg of linearized plasmid DNA (carrying ctRPF2-FLAG-TEV-ProtA (ctRPF2-FtpA), ctRRS1-FtpA or ctSYO1-FtpA) and incubated on ice for 20 min. Then, 750 μl of STC/PEG solution were added, and after 10 min of incubation at room temperature (~22 °C) with gentle shaking on a turning wheel, the protoplasts were plated on CCM agar supplemented with 0.8 M sorbitol and the selection marker (terbinafine hydrochloride) used at a final concentration of 0.5 μg ml^−1^. Plates were incubated at 50–55 °C for 2–3 days. Transformants were tested for expression of the affinity-tagged fusion proteins, and positive transformants were selected and induced to sporulate. Spores were stored in the Hurt laboratory’s *C. thermophilum* collection at −80 °C. For isolation of 5S RNP complexes, *C. thermophilum* transformants containing the desired 5S RNP-related factors (Rpf2, Rrs1 and Syo1) FtpA-tagged were grown in CCM at 50–55 °C for 12–24 h, then the grown mycelium was collected for affinity purification (described below).

### In vivo reconstitution of 5S RNP complexes from yeast

Yeast W303 wild-type strain was co-transformed with three multicopy plasmids by the lithium acetate/single-stranded carrier DNA/polyethylene glycol method as previously described^48^. Each plasmid with distinct auxotrophic markers, which all together carried *scSYO1*, *scRPF2*, *scRRS1*, *RPL5* (*scuL18*) and *RPL11* (sc*uL5*) constructs, was under the control of the *GAL* promoter. Split-tag affinity purifications based on ProtA and FLAG epitopes yielded either *sc*Syo1–*sc*Rrs1 or *sc*uL5–*sc*Rrs1 pairs, which were used for affinity purification. To detect the components of the reconstituted hexameric 5S RNP by western blotting, we used combinations of constructs, some of which contained the additional HA tag. To distinguish the 5S RNP that assembled into early nucleolar pre-60S particles by negative-stain EM analysis, we used the plasmid containing uL18 fused to a 3×GFP tag. Positive-tested transformants were grown in medium containing galactose (YPGal: 10 g of yeast extract, 20 g of peptone and 20 g of galactose, per liter, pH 5.5) at 30 °C until reaching an optical density (OD) of 2, then centrifuged, and pellets were kept frozen at −20 °C until tandem affinity purification (described below).

### In vivo reconstitution of the *ct–sc* 5S RNP chimera

Yeast W303 wild-type strain was co-transformed with plasmids carrying ProtA-TEV-*ctSYO1*, *ctRPF2* and FLAG-*ctRRS1*, all under the control of the *GAL* promoter. Positive-tested transformants were grown in medium containing galactose (YPGal) at 30 °C until reaching an OD of 2, then cells were collected by centrifugation and kept frozen at −20 °C until tandem affinity purification (described below).

### In vivo reconstitution of the *hs–sc* 5S RNP chimera

The yeast shuffle strain *rpf2Δ* complemented by human *hsRPF2* was co-transformed with plasmids carrying ProtA-TEV-*hsuL5*, FLAG-*hsRRS1*, *hsuL18* and *hsSYO1*, with all of the human genes under the control of the *GAL* promoter. Positive-tested transformants were grown in medium containing galactose (YPGal) at 30 °C until reaching an OD of 2, then centrifuged, and pellets were kept frozen at −20 °C until tandem affinity purification (described below).

### In vivo reconstitution of the Mdm2–5S RNP complex

Yeast W303 wild-type strain was co-transformed with plasmids carrying *hsuL18*-TEV-ProtA, *hsMdm2-*FLAG and *hsuL5*, all under the control of the *GAL* promoter. Positive-tested transformants were grown in medium containing galactose (YPGal) at 30 °C until reaching an OD of 2, then centrifuged, and pellets were kept frozen at −20 °C until tandem affinity purification (described below).

### Depletion of 5S RNP in pre-60S ribosomal particles

Genomic tagging of pre-60S ribosome-related factors (Nsa1, Nsa3 and Yvh1) was performed using the lithium acetate procedure with PCR-based DNA cassettes as previously described for yeast^49^. The yeast W303 wild-type strain carrying the genomically integrated *GAL*::*HA-RPF2*, *GAL*::*HA-RRS1* or *GAL*::*HA-UL18*, together with either *NSA1*-FtpA or *NSA3*-FtpA, was grown in medium containing galactose (YPGal) at 30 °C until reaching an OD of 0.45, then shifted to glucose (YPGlu: yeast extract, 20 g of peptone and 20 g of glucose, per liter, pH 5.5) at 30 °C for 6 h to deplete the entire 5S RNP, or kept in YPGal for non-depletion conditions (cells were collected at a final OD of approximately 2). Pellets were kept frozen at −20 °C until tandem affinity purification (described below).

### Tandem affinity purification

Purification of bait proteins from yeast or *C. thermophilum* was performed in NB-HEPES buffer (20 mM HEPES, pH 7.5, 150 mM NaCl, 50 mM K(OAc), 2 mM Mg(OAc)_2_, 1 mM DTT, 5% glycerol and 0.1% (vol/vol) IGEPAL CA-630). As described before, yeast cell pellets^50^ or mycelium^47^ were lysed by cryogenic grinding in a cell mill (Retsch, MM 400) using the NB-HEPES buffer with SIGMAFAST Complete Protease Inhibitor Cocktail (Sigma–Aldrich) added at a ratio of approximately 1 ml of buffer per gram of cells. The lysate was cleared (20,000 rpm at 4 °C for 20 min), and bait proteins were affinity purified from the supernatant using 250 μl of IgG Sepharose suspension (IgG Sepharose 6 Fast Flow, Sigma–Aldrich). After two washes with NB-HEPES buffer, proteins were eluted by TEV protease at 16 °C for 2 h and subsequently incubated with 50 μl of ANTI-FLAG M2 Affinity Gel (Sigma–Aldrich) at 4 °C for 2 h. Bound proteins were eluted using 100 μg ml^−1^ FLAG peptide (Sigma–Aldrich) at 4 °C for 45 min. FLAG eluates were loaded on 4–12% Bis-Tris polyacrylamide gradient gels (NuPAGE, Invitrogen) and stained with Coomassie. Western blots were performed according to standard procedures with the following antibody dilutions: anti-HA, 1:1,000; anti-pA, 1:3,000; anti-FLAG, 1:2,000; and anti-RPL5, 1:1,000.

### Sucrose gradient centrifugation

Final eluates obtained from the tandem affinity purifications were loaded onto a linear 5–30% (w/v) sucrose gradient with the same buffer used for purification. Samples were centrifuged at 130,000 × *g* at 4 °C for 14 h in an SW 40 rotor (Beckman Coulter). The sucrose gradients were fractionated, and each fraction was either precipitated with 10% trichloroacetic acid (TCA) or analyzed by negative-stain EM. TCA-precipitated proteins were resuspended in SDS sample buffer and analyzed by NuPAGE and Coomassie staining.

### In vitro binding assay (5S RNP–pre-60S ribosomes)

Pre-60S particles depleted of the endogenous 5S RNP (described above) were incubated with purified hexameric 5S RNP complexes that had been affinity purified from strains overexpressing 5S RNP proteins (see above). FLAG eluates derived from the depleted pre-60S particles and the purified 5S RNP were mixed in different molar ratios and incubated at room temperature for 45 min on a turning wheel, before the mixtures were analyzed by sucrose gradient centrifugation. Final FLAG eluates or selected fractions from the sucrose gradients were analyzed by single-quadrupole mass spectrometry (SQ-MS) at FingerPrints Proteomics (University of Dundee, UK). Co-precipitating proteins were identified by one-dimensional nanoscale liquid chromatography–tandem mass spectrometry with electrospray ionization (nLC–ESI–MS/MS) using MaxQuant software^51^. Intensity-based absolute quantification (iBAQ) values of label-free quantification are shown in the Source Data Excel file. Single-band identification by mass spectrometry was conducted in-house at the BZH mass spectrometry service (Heidelberg University, Germany).

### In vivo binding assay (5S RNP–pre-60S ribosomes)

Yeast strain W303 carrying *GAL*::*HA-RPF2* and *GAL*::*HA-RRS1* was transformed with single-copy plasmids that contained either wild-type or mutant *RPF2* and *RRS1* constructs that were under the control of their endogenous promoters. Ectopically expressed Rrs1ΔC or Rpf2ΔC tagged with FLAG-TEV-ProtA was used as bait for affinity purification. Cells were grown, shifted from galactose-containing to glucose-containing medium to repress *GAL*::*HA-RPF2* and *GAL*::*HA-RRS1* and collected after 6 h. Final eluates from tandem affinity purifications were loaded onto 5–30% sucrose gradients. After centrifugation, fractions from the sucrose gradients were analyzed by SDS–polyacrylamide gel electrophoresis (SDS–PAGE) and standard Coomassie staining.

### XL-MS of the 5S RNP complexes

Purified 5S RNPs were crosslinked with isotopically coded disuccinimidyl suberate (DSS-H12/D12, Creative Molecules) in 0.5 mM increments to a final DSS concentration of 4 mM. The resulting crosslinked proteins were digested, and crosslinked peptides were enriched by gel filtration as previously described^52^. Peptides were analyzed by LC–MS/MS on an Orbitrap Fusion Lumos mass spectrometer (Thermo) using a top-ten strategy with MS1 scans on the Orbitrap (*R* = 120,000; *m*/*z* range, 375–1,600), followed by fragmentation by collision-induced dissociation (isolation window, 0.8 *m*/*z*; collision energy, 35%; activation time, 10 ms; activation Q, 0.25) and MS2 detection on the ion trap of ions with charge states of 3–7. Raw files were analyzed, and false discovery rates were estimated using xQuest^53^ and xProphet^54^ against a sequence database containing the included 5S RNP proteins and Mdm2 where applicable. Only first-ranked crosslinks with a false discovery rate of ≤0.05 were considered. Crosslinks were visualized using xiNet^45^, Xlink Analyzer^55^ and UCSF Chimera^56^.

### Negative-stain EM and image analysis

Negative staining, data collection and processing were performed as previously described^57^. For 2D classification, particles were selected using the Boxer program in EMAN2^58^. Image processing was performed using the IMAGIC-4D package^59^. Particles were bandpass filtered, normalized in their gray value distribution and mass centered. Approximate numbers of total particles for 2D classification and averaging are as follows: Nsa3-FtpA (wild type), 7,747; Nsa3-FtpA (depleted), 10,202; Nsa3-FtpA (depleted + 5S RNP), 13,050; and Nsa3-FtpA (depleted + 5S RNP–3×GFP), 13,532.

### Cryo-EM and image processing

Holey gold grids (R 1.2/1.3 QUANTIFOIL) and holey carbon support grids (R3/3 with 2 nm carbon support, QUANTIFOIL) were glow discharged at 2.1 × 10^−1^ Torr for 20 s. The samples (3.5 µl) were applied onto the grids at 4 °C and 95% humidity using a Vitrobot Mark IV (FEI) and plunge frozen in liquid ethane. The pre-60S sample was incubated on the carbon support grids for 45 s before plunge freezing, whereas the 5S RNP samples were frozen without incubation time on grids without carbon support. Data of the different 5S RNP samples were collected on a Titan Krios operated at 300 kV and equipped with a K2 Summit direct electron detector operated in counting mode (micrograph pixel size, 1.059 Å), and the pre-60S data were collected on a Titan Krios operated at 300 kV and equipped with a Falcon II direct electron detector (micrograph pixel size, 1.084 Å). All collections were performed according to low dose practice and a target defocus. We performed two collections of the *ct* hexameric 5S RNP sample (dataset 1, 1,738 and 5,302 micrographs), two collections of the chimeric *ct–sc* 5S RNP sample (dataset 2, 1,087 and 1,032 micrographs), one collection of the human 5S RNP–Mdm2 sample (dataset 3, 2,504 micrographs), and one collection of the pre-60S containing the human 5S RNP (dataset 4, 4,660 micrographs). Gctf^60^ and CTFFIND4^61^ were used to estimate the contrast transfer function (CTF) parameters. The micrographs were manually inspected, and 6,550, 1,583, 2,454 and 3,937 micrographs for datasets 1, 2, 3 and 4, respectively, were used for further processing (Table 1). crYOLO^62^ was used for particle picking. For datasets 1, 2 and 3, particles on 43, 123 and 11 micrographs, respectively, were manually picked and used to generate individual models for each dataset. For dataset 4, JANNI denoising and the crYOLO general model were used. The 2D classification of datasets 1, 2 and 4 was performed with cryoSPARC^63^, and the 2D classification of dataset 3 was performed with RELION 3.1^64,65^. Dataset 4 was refined against EMDB-6615^10^, and subsequent three-dimensional (3D) classifications were performed with RELION 3.1. The final homogeneous refinement and CTF parameter refinement were performed in cryoSPARC (see also Extended Data Fig. 3g). Good particles after 2D classification for datasets 1 and 2 and a subset of particles for dataset 3 were used for ab initio reconstruction with three classes in cryoSPARC. The corresponding ab initio volumes were used as references for heterogeneous refinement with all picked particles. Subsequent heterogeneous refinements, non-uniform refinement^66^ and local filtering were performed with cryoSPARC (see Extended Data Figs. 1d and 2d). Dataset 3 was further processed with RELION 3.1 using the 5S RNP shaped ab initio volume (cryoSPARC) as initial reference. The final 3D refinement was performed either with a mask around the 5S RNP or unmasked (see Extended Data Fig. 7c).

### Model building and refinement

For hexameric *ct*5S RNP, homology models of uL5, uL18, Rrs1 and Rpf2 from *C. thermophilum* were generated with SWISS-MODEL^67^ using *S. cerevisiae* models of the Nog2 pre-60S particle as reference (PDB ID 3JCT). The *C. thermophilum* homology models, the crystal structure of *ct*Syo1 (PDB ID 4GMO) and the yeast 5S rRNA (PDB ID 3JCT) were rigid body fitted into the density of the hexameric *C. thermophilum* 5S RNP density using Chimera^56^. The fitted models were analyzed and manually refined with Coot^68^. The model of the *ct*5S rRNA was generated by exchange of the *S. cerevisiae* 5S rRNA model sequence with the *ct*5S rRNA sequence and manual correction of the bases according to the density in Coot. For human MDM2–5S RNP, the models of *hs*uL18, *hs*uL5 (PDB ID 6ZM7), the yeast 5S rRNA (PDB ID 3JCT) and the crystal structure of human MDM2–uL5 (4XXB) were rigid body fitted into the density of the human MDM2–5S RNP, and the resulting model was manually corrected in Coot. The side chains for uL18, uL5 and MDM2 were removed. The models of the hexameric *ct*5S RNP and the human MDM2–5S RNP were real-space refined with Phenix^69,70^, and results were inspected in Coot. ChimeraX^71^ was used to visualize cryo-EM densities and molecular models and was used for figure preparation.

### Northern blot analysis

RNA from FLAG eluates was extracted with phenol/chloroform/isoamyl alcohol and recovered by precipitation with ethanol. Pellets were resuspended and resolved on 6% polyacrylamide/8.3 M urea gels stained with SYBR Green II (Sigma–Aldrich). For northern blot analysis, after transfer to membranes (Hybond-N+, GE Healthcare), the following probes were 5′-labeled with ^32^P using standard procedures: *sc*5S rRNA (5′-CTACTCGGTCAGGCTC-3′) and *ct*5S rRNA (5′-TCAGTGGCTTGTCTATGG-3′).

### EMSAs

Plasmids for the production of tRNA^Phe^ and 25S rRNA^(H81–H87)^ were linearized to use as template for RNA in vitro synthesis. We used the HiScribe T7 High Yield RNA Synthesis Kit (New England Biolabs) for high-specific-activity radiolabeled RNA probe synthesis with [α-^32^P] UTP 3,000 Ci mmol^−1^, 10 mCi ml^−1^ (Hartmann Analytic) according to the manufacturer’s instructions. The binding reactions were performed with a fixed amount of radiolabeled RNA and serial dilutions (1:3 factor) of the 5S RNP complexes and incubated at room temperature for 20 min in a final volume of 10 μl, containing the same buffer used for protein purification. BSA was added to each reaction as a non-specific competitor. Separation was performed at 4 °C on a 2.5% acrylamide native gel. 5S RNP complexes from yeast were isolated by tandem affinity purification as described above. For the 5S RNP complex purification lacking Syo1, we used the SYO1-deleted strain as background, pA-TEV-uL18 as first bait and FLAG-Rrs1 as second bait. For the complex carrying the double truncation (Rpf2ΔC/Rrs1ΔC) and Syo1, we used the RRS1 shuffle strain complemented with *RRS1Δ*C as background, pA-TEV-Syo1 as first bait and FLAG-Rpf2ΔC as second bait. For the complex with the double truncation (Rpf2ΔC/Rrs1ΔC) but lacking Syo1, we used the latter-mentioned baits, but used the SYO1-deleted RRS1 shuffle strain as source.

### Yeast two-hybrid assay

For the analysis of two-hybrid interactions, *hs*Mdm2 and *hs*uL18 (wild-type and truncation constructs) were C-terminally fused to the DNA-binding domain and the transcription activation domain, respectively, of the *GAL4* transcription factor from *S. cerevisiae*. The yeast two-hybrid reporter strain PJ69-4 was transformed with the pGBKT7 and pGADT7 plasmids that carry the corresponding pair of bait and prey constructs, respectively. Transformants were selected and pre-grown on SC−Leu−Trp plates before dot-spotting them onto SC−Leu−Trp−His plates to score the two-hybrid interaction after growth at 30 °C for 3 days.

### Reporting summary

Further information on research design is available in the Nature Portfolio Reporting Summary linked to this article.

## Online content

Any methods, additional references, Nature Portfolio reporting summaries, source data, extended data, supplementary information, acknowledgements, peer review information; details of author contributions and competing interests; and statements of data and code availability are available at 10.1038/s41594-023-01006-7.

## Supplementary information

Supplementary Information Negative-stain EM analysis of pre-60S particles reconstituted with the 5S RNP. Negative-stain EM of the pre-60S particles before and after 5S RNP-depletion, and after reconstitution with the yeast 5S RNP containing uL18 untagged or tagged with three GFP moieties (3×GFP). All 2D class averages obtained for this analysis are depicted. Characteristic 2D classes boxed with a red square are further shown in Fig. 3b. Scale bar, 20 nm.

Reporting Summary

Supplementary Table 1 Strains, materials and reagents.

## Data Availability

The 3D cryo-EM maps have been deposited at the Electron Microscopy Data Bank (EMDB) under the accession numbers EMD-13134, EMD-16036, EMD-16037, EMD-16038 and EMD-16040. The atomic models of the hexameric 5S RNP from *C. thermophilum* and the human MDM2–5S RNP have been deposited at the PDB under the IDs 7OZS and 8BGU, respectively. The XL-MS and SQ-MS output data files for Figs. 1f, 3a and 6a are provided with this paper in the Source Data Excel file, and the corresponding mass spectrometry raw data have been deposited to the ProteomeXchange Consortium via the PRIDE^72^ partner repository with the dataset identifiers PXD040087 and PXD040306, respectively. Unprocessed and uncropped images, as well as numerical raw data, in Figs. 1c, 4c, 5a and 6c,d, and Extended Data Figs. 1a and 5b are provided with this paper as Source Data files.

## Source data

Source Data Figs. 1, 3 and 6 and Extended Data Fig. 5 Fig. 1f XL-MS output file.
Source Data Fig. 1 Northern blots, uncropped images.
Source Data Fig. 4 Northern blots, uncropped images.
Source Data Fig. 5 Methylene blue staining, uncropped images.
Source Data Fig. 6 Western blot, methylene blue staining and uncropped images.
Source Data Extended Data Fig. 1 Methylene blue staining, uncropped images.
Source Data Extended Data Fig. 4 Methylene blue staining, uncropped images.
Source Data Extended Data Fig. 5 Methylene blue staining, western blot and uncropped images.
