# Supplementary material for: Structure of nascent 5S RNPs at the crossroad between ribosome assembly and MDM2–p53 pathways
Source: Nat Struct Mol Biol. 2023 Jun 8;30(8):1119–31. doi: 10.1038/s41594-023-01006-7 (PMC10442235; doi:10.1038/s41594-023-01006-7)
Supplement: Supplementary file 1 — Negative-stain EM analysis of pre-60S particles reconstituted with the 5S RNP. Negative-stain EM of the pre-60S particles before and after 5S RNP-depletion, and after reconstitution with the yeast 5S RNP containing uL18 untagged or tagged with three GFP moieties (3×GFP). All 2D class averages obtained for this analysis are depicted. Characteristic 2D classes boxed with a red square are further shown in Fig. 3b. Scale bar, 20 nm. [file 41594_2023_1006_MOESM1_ESM.pdf]

nature structural & molecular biology

Article https://doi.org/10.1038/s41594-023-01006-7

Structure of nascent 5S RNPs at the crossroad between ribosome assembly and MDM2–p53 pathways

In the format provided by the authors and unedited

Nsa3-FtpA
(Non-Depleted)

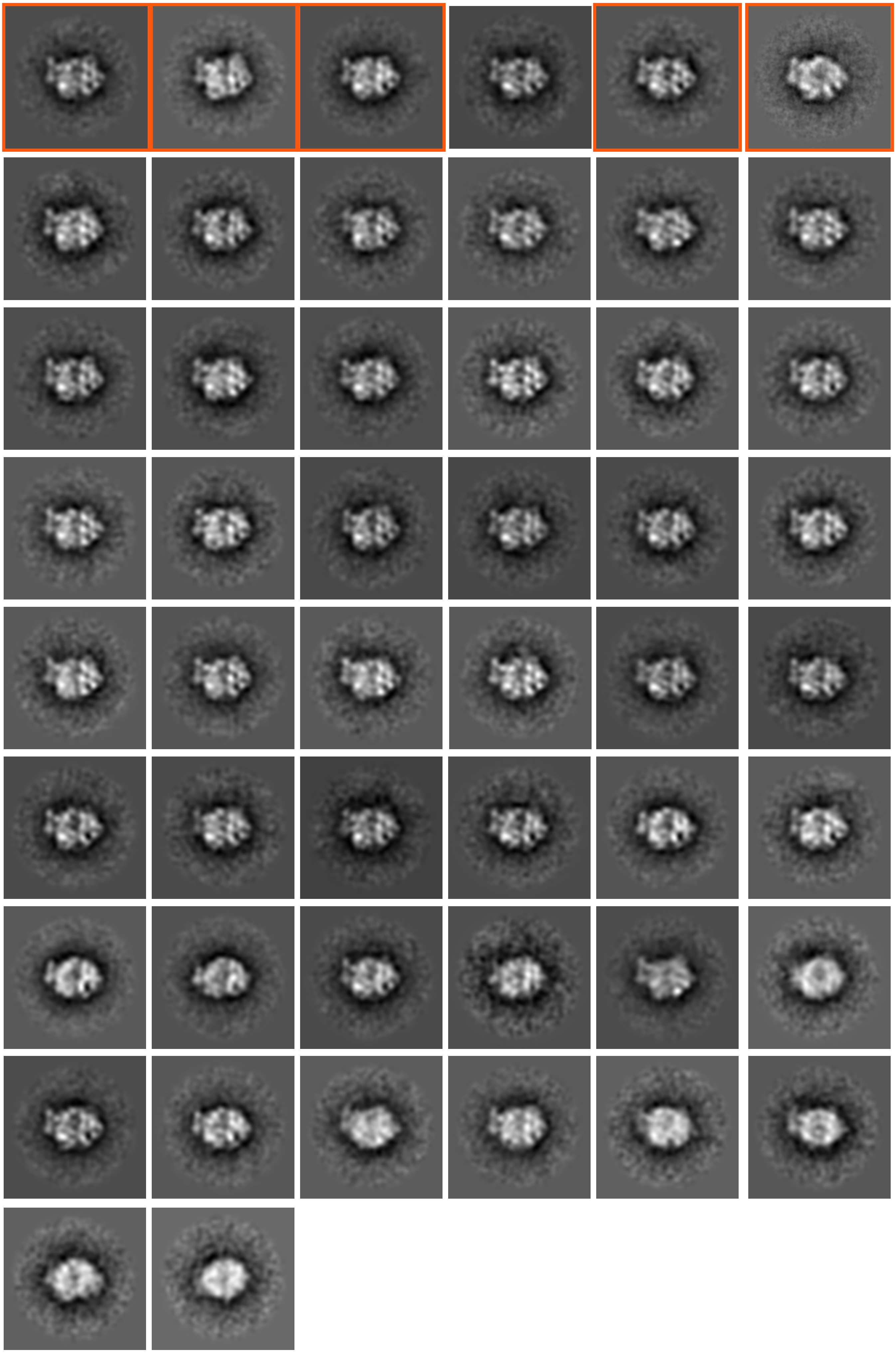

Nsa3-FtpA
(5S RNP-depleted)

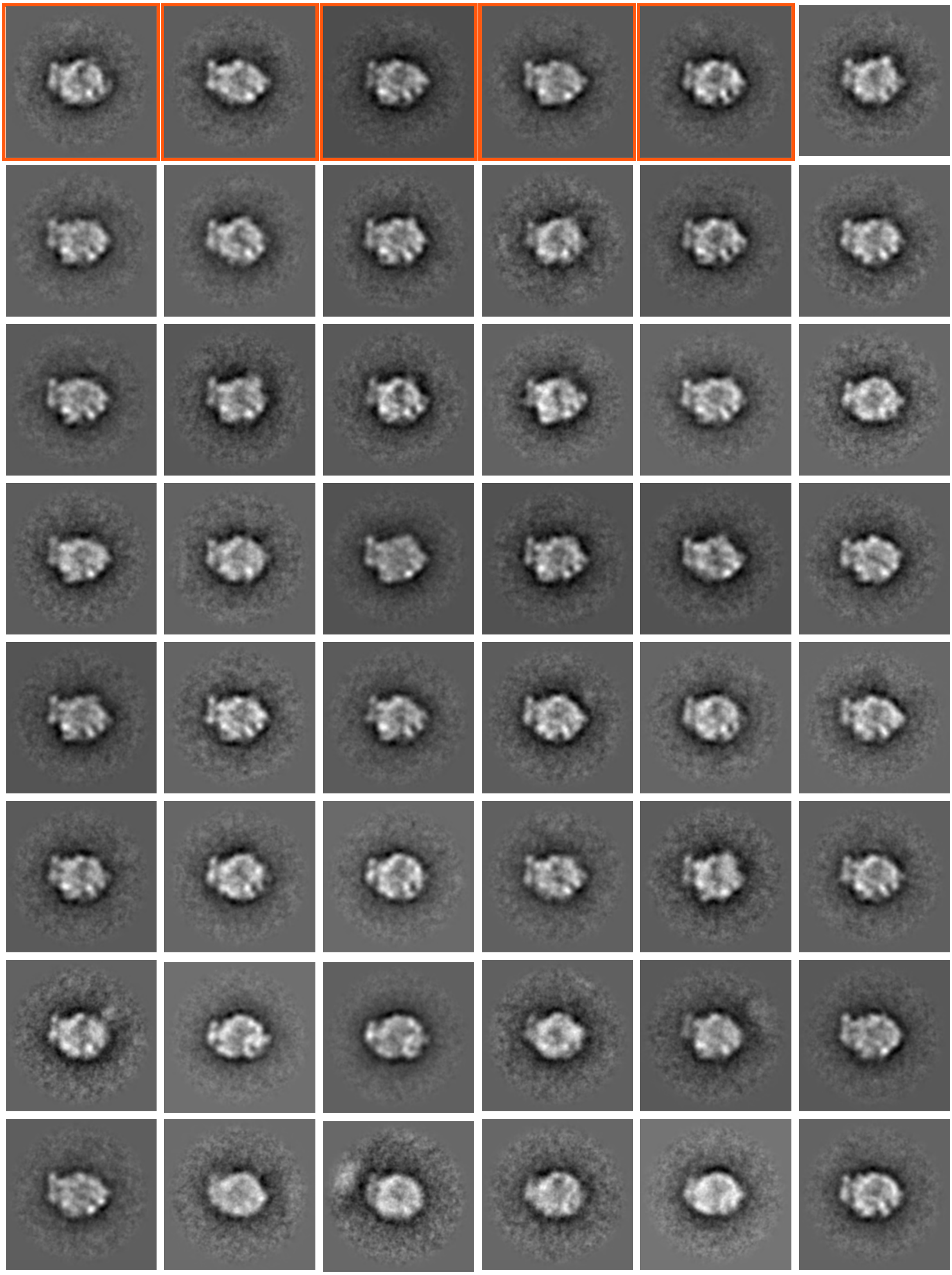

Nsa3-FtpA
(5S RNP-depleted)
+ 5SRNP

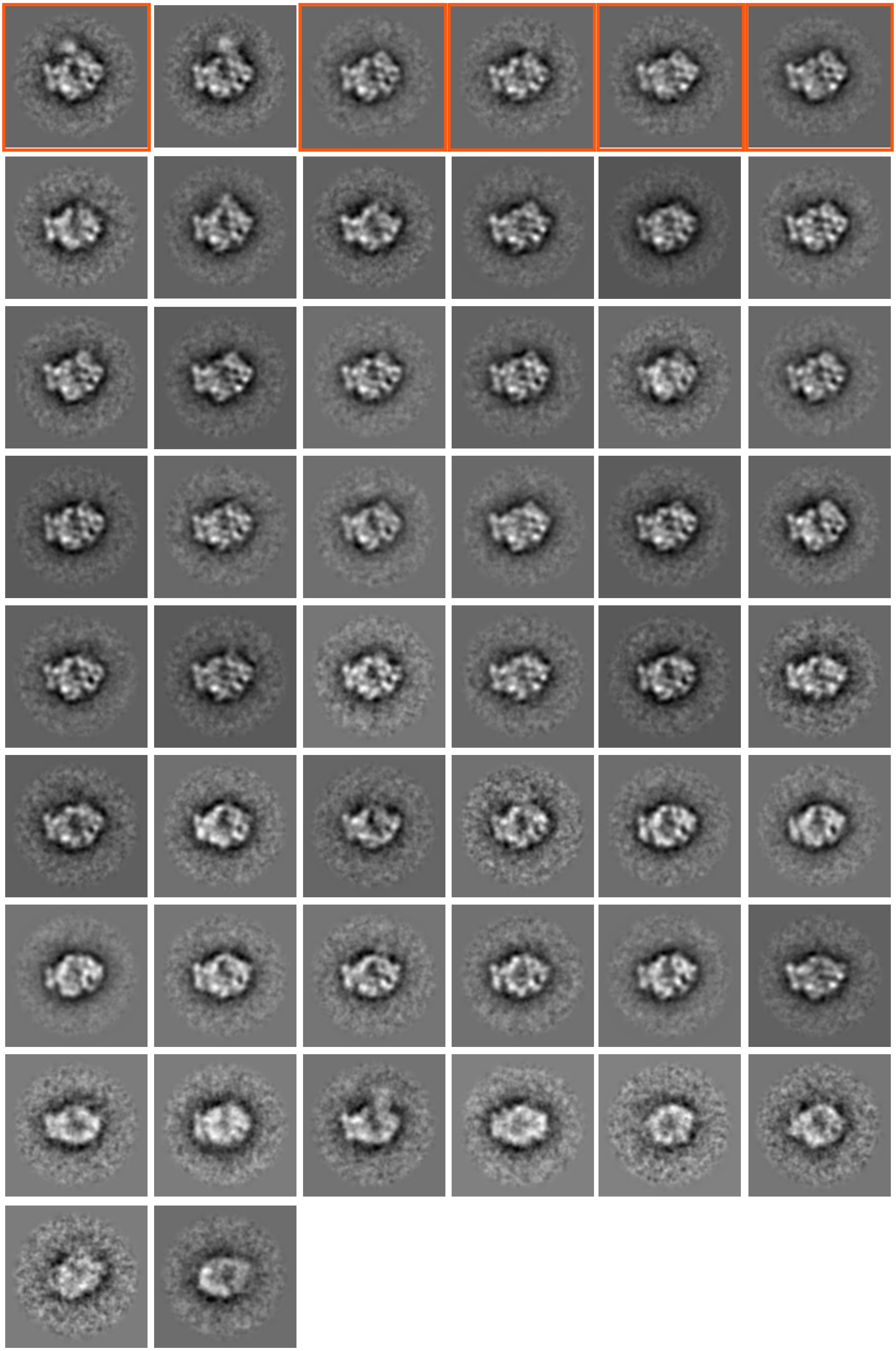

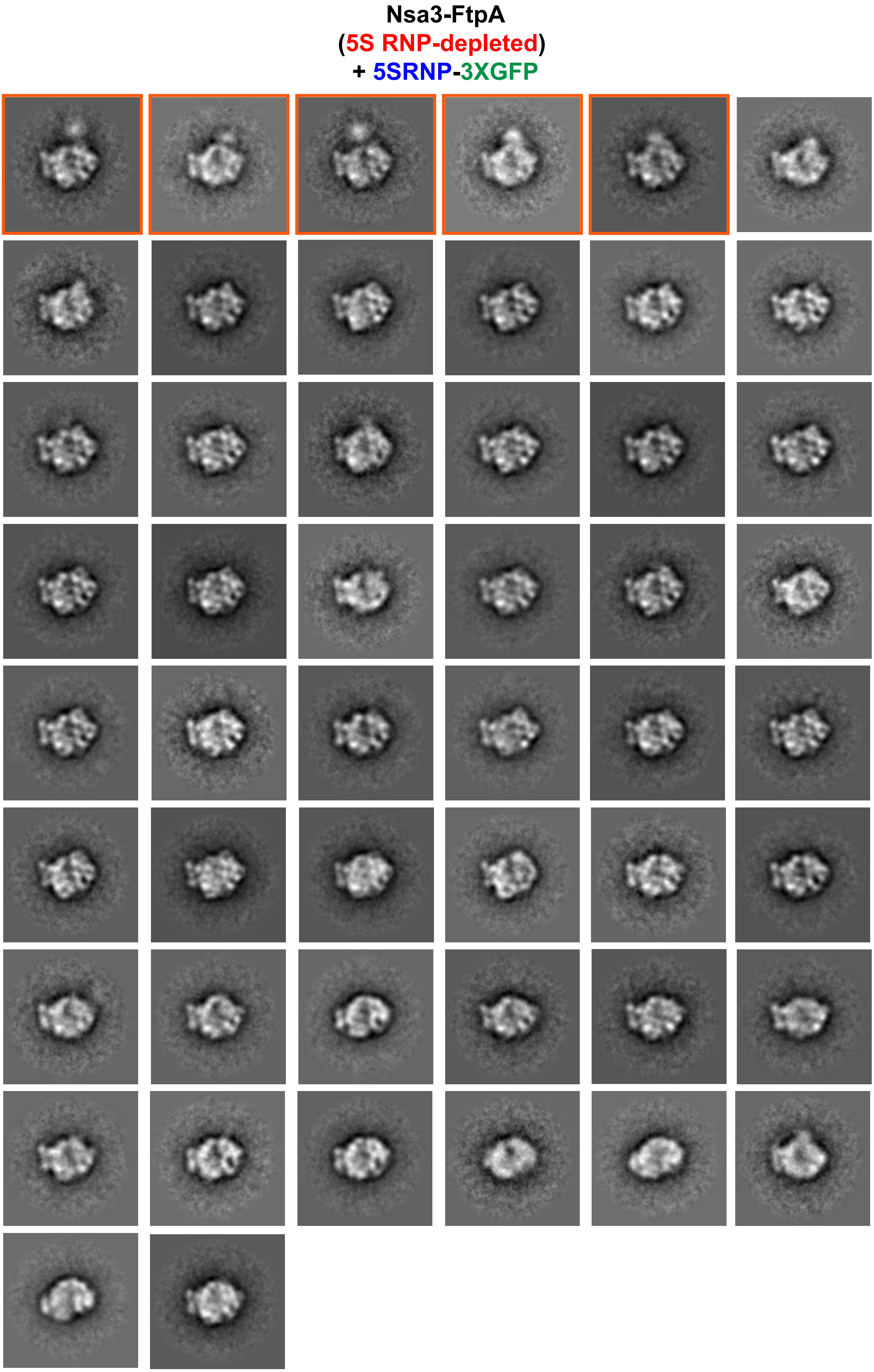

Nsa3-FtpA
(5S RNP-depleted)
+ 5SRNP-3XGFP
