# Supplementary material for: Structure of nascent 5S RNPs at the crossroad between ribosome assembly and MDM2–p53 pathways
Source: Nat Struct Mol Biol. 2023 Jun 8;30(8):1119–31. doi: 10.1038/s41594-023-01006-7 (PMC10442235; doi:10.1038/s41594-023-01006-7)
Supplement: Supplementary file 2 — Reporting Summary [file 41594_2023_1006_MOESM2_ESM.pdf]

nature portfolio

Corresponding author(s): Ed Hurt, Roland Beckman

Last updated by author(s): Oct 29, 2022

Reporting Summary

Nature Portfolio wishes to improve the reproducibility of the work that we publish. This form provides structure for consistency and transparency in reporting. For further information on Nature Portfolio policies, see our Editorial Policies and the Editorial Policy Checklist.

Statistics

For all statistical analyses, confirm that the following items are present in the figure legend, table legend, main text, or Methods section.

n/a	Confirmed	
☐	☒	The exact sample size (*n*) for each experimental group/condition, given as a discrete number and unit of measurement
☐	☒	A statement on whether measurements were taken from distinct samples or whether the same sample was measured repeatedly
☒	☐	The statistical test(s) used AND whether they are one- or two-sided<br>*Only common tests should be described solely by name; describe more complex techniques in the Methods section.*
☒	☐	A description of all covariates tested
☒	☐	A description of any assumptions or corrections, such as tests of normality and adjustment for multiple comparisons
☐	☒	A full description of the statistical parameters including central tendency (e.g. means) or other basic estimates (e.g. regression coefficient) AND variation (e.g. standard deviation) or associated estimates of uncertainty (e.g. confidence intervals)
☒	☐	For null hypothesis testing, the test statistic (e.g. *F*, *t*, *r*) with confidence intervals, effect sizes, degrees of freedom and *P* value noted<br>*Give P values as exact values whenever suitable.*
☒	☐	For Bayesian analysis, information on the choice of priors and Markov chain Monte Carlo settings
☒	☐	For hierarchical and complex designs, identification of the appropriate level for tests and full reporting of outcomes
☒	☐	Estimates of effect sizes (e.g. Cohen's *d*, Pearson's *r*), indicating how they were calculated

Our web collection on statistics for biologists contains articles on many of the points above.

Software and code

Policy information about availability of computer code

Data collection	EPU
Data analysis	xQuest, xProphet, xiNet, Xlink Analyzer, EMAN2, IMAGIC-4D, RNAfold, crYOLO, REL ION 3.1, cryoSPARC v2.15.0 and v3 .2.0, UCSF Chimera 1.14, ChimeraX 1.1, WinCoot 0. 9.4.1, Phenix 1.19.2

For manuscripts utilizing custom algorithms or software that are central to the research but not yet described in published literature, software must be made available to editors and reviewers. We strongly encourage code deposition in a community repository (e.g. GitHub). See the Nature Portfolio guidelines for submitting code & software for further information.

Data

Policy information about availability of data

All manuscripts must include a data availability statement. This statement should provide the following information, where applicable:

- Accession codes, unique identifiers, or web links for publicly available datasets
- A description of any restrictions on data availability
- For clinical datasets or third party data, please ensure that the statement adheres to our policy

The 3D cryo-EM maps have been deposited at the Electron Microscopy Data Bank (EMDB) under the accession numbers EMD-13134, EMD-16036, EMD-16037, EMD-16038 and EMD-16040. The atomic models of the hexameric 5S RNP from C. thermophilum and the human MDM2-5S RNP have been deposited at the Protein Data Bank under the accession codes PDB: 7OZS and PDB: 8BGU, respectively. The Crosslink-MS and SQ-MS output data files for Fig.1f, 3a and 6a are provided with

this paper as Supplementary Data. Unprocessed and uncropped images, as well as numerical raw data, in Figures 1c, 4c, 5a, 6c and 6d, and Extended Data Figures 1a and 5b, are provided with this paper as Source Data files.

Human research participants

Policy information about studies involving human research participants and Sex and Gender in Research.

Reporting on sex and gender	*Use the terms sex (biological attribute) and gender (shaped by social and cultural circumstances) carefully in order to avoid confusing both terms. Indicate if findings apply to only one sex or gender; describe whether sex and gender were considered in study design whether sex and/or gender was determined based on self-reporting or assigned and methods used. Provide in the source data disaggregated sex and gender data where this information has been collected, and consent has been obtained for sharing of individual-level data; provide overall numbers in this Reporting Summary. Please state if this information has not been collected. Report sex- and gender-based analyses where performed, justify reasons for lack of sex- and gender-based analysis.*
Population characteristics	*Describe the covariate-relevant population characteristics of the human research participants (e.g. age, genotypic information, past and current diagnosis and treatment categories). If you filled out the behavioural & social sciences study design questions and have nothing to add here, write "See above."*
Recruitment	*Describe how participants were recruited. Outline any potential self-selection bias or other biases that may be present and how these are likely to impact results.*
Ethics oversight	*Identify the organization(s) that approved the study protocol.*

Note that full information on the approval of the study protocol must also be provided in the manuscript.

Field-specific reporting

Please select the one below that is the best fit for your research. If you are not sure, read the appropriate sections before making your selection.

☒ Life sciences ☐ Behavioural & social sciences ☐ Ecological, evolutionary & environmental sciences

For a reference copy of the document with all sections, see nature.com/documents/nr-reporting-summary-flat.pdf

Life sciences study design

All studies must disclose on these points even when the disclosure is negative.

Sample size	No statistical methods were used to predetermine sample size because in most of our experiments no statistical analysis were required. Sample size for growth curves experiments (n=3) was selected based on variance observed in prior experiments of a similar nature, as well as practical considerations. For cryo-EM analysis the sample size was determined by the limited access to the microscope.
Data exclusions	During cryo-EM processing, bad particle images were removed. A description can be found in the method section and detailed processing schemes of the different cryo-EM datasets are shown as Extended Data Figures.
Replication	All experiments were repeated at least three times with reproducible results, except the radioactive EMSA assay that was repeated twice also with similar results.
Randomization	No randomization was necessary for this study because investigators were purifying proteins, DNA and RNA for structure determination and biochemical assays under well controlled conditions. No human or animal subjects were used in the study. Randomization is not generally used in this field.
Blinding	Blinding was not necessary because the samples and results did not require subjective judgment or interpretation. Blinding is not typically used in the field.

Reporting for specific materials, systems and methods

We require information from authors about some types of materials, experimental systems and methods used in many studies. Here, indicate whether each material, system or method listed is relevant to your study. If you are not sure if a list item applies to your research, read the appropriate section before selecting a response.

Materials & experimental systems

n/a	Involved in the study
☐	☒ Antibodies
☐	☒ Eukaryotic cell lines
☒	☐ Palaeontology and archaeology
☒	☐ Animals and other organisms
☒	☐ Clinical data
☒	☐ Dual use research of concern

Methods

n/a	Involved in the study
☒	☐ ChIP-seq
☒	☐ Flow cytometry
☒	☐ MRI-based neuroimaging

Antibodies

Antibodies used

Anti-FLAG-Peroxidase, Sigma-Aldrich (Cat# H7425)
Anti-Protein A, Sigma-Aldrich (Cat# P3775)
Anti-HA, Abcam (Cat# ab18181)
RPL5 Antibody, Cell Signaling Technology (Cat# 14568)

Validation

The antibodies used in this study were rigorously validated for Western Blotting according to the manufacterers's websites. Also we have validated them in our own lab while working with yeast and and C. thermophilum, observing similar Molecular Weight to the expected target and by combination with Mass Spectrometry analysis of the correspondent target protein bands. Additionally, these antibodies have been commonly used in the field, and by our own lab, for many years with reliable and reproducible outcome.

Eukaryotic cell lines

Policy information about cell lines and Sex and Gender in Research

Cell line source(s)

S. cerevisiae: W303 wild type strain (Thomas & Rothstein, 1989)
S. cerevisiae: W303; SYO1::natNT2 (Kressler et al., 2012)
S. cerevisiae: W303; SYO1::natNT2; RPL5::His3MX6, pRS316-scUL18 (Kressler et al., 2012)
S. cerevisiae: W303; RPF2::natNT2; pRS316-RPF2 (This study)
S. cerevisiae: W303; RRS1::natNT2, pRS316-RRS1 (This study)
S. cerevisiae: W303; SYO1::natNT2, RPF2::His3MX6, pRS316-RPF2 (This study)
S. cerevisiae: w303, PNSA1-NSA1-FtpA:natNT2 (This study)
S. cerevisiae: w303, PNSA3-NSA3-FtpA:natNT2 (This study)
S. cerevisiae: w303, PYVH1-YVH1-FtpA:klURA3 (This study)
S. cerevisiae: w303, PNSA1-NSA1-FtpA:natNT2, His3MX6:PGAL-HA-RPF2 (This study)
S. cerevisiae: w303, PNSA1-NSA1-FtpA:natNT2, His3MX6:PGAL-HA-RRS1 (This study)
S. cerevisiae: w303, PNSA1-NSA1-FtpA:natNT2, His3MX6:PGAL-HA-scUL18 (This study)
S. cerevisiae: w303, PNSA3-NSA3-FtpA:natNT2, His3MX6:PGAL-HA-RPF2 (This study)
S. cerevisiae: w303, PNSA3-NSA3-FtpA:natNT2, His3MX6:PGAL-HA-RRS1 (This study)
S. cerevisiae: w303, PNSA3-NSA3-FtpA:natNT2, His3MX6:PGAL-HA-scUL18 (This study)
S. cerevisiae: w303, His3MX6:PGAL-HA-RPF2, His3MX6:PGAL-HA-RRS1 (This study)
S. cerevisiae: Trp1–901, Leu2–3112 Ura3–52, His3-200, gal4Δ, gal80Δ, LYS2::GAL1–HIS3, GAL2-ADE2, met2::GAL7-lacZ (James et al., 1996)
C. thermophilum: PACTIN-pA-TEV-FLAG-ctRPF2 (CTHT_0061110) (This study)
C. thermophilum: PACTIN-pA-TEV-FLAG-ctRRS1 (CTHT_0057260) (This study)
C. thermophilum: PACTIN-pA-TEV-FLAG-ctSYO1 (CTHT_0033460) (This study)

Authentication

None of the cell lines used were authenticated

Mycoplasma contamination

N/A

Commonly misidentified lines
(See ICLAC register)

N/A
