# Supplementary material for: Structure of nascent 5S RNPs at the crossroad between ribosome assembly and MDM2–p53 pathways
Source: Nat Struct Mol Biol. 2023 Jun 8;30(8):1119–31. doi: 10.1038/s41594-023-01006-7 (PMC10442235; doi:10.1038/s41594-023-01006-7)
Supplement: Supplementary file 3 — Strains, materials and reagents. [file 41594_2023_1006_MOESM3_ESM.pdf]

Supplementary Table 1: Strains, materials and reagents

	SOURCE	IDENTIFIER
**Antibodies**		
Anti-FLAG-Peroxidase	Sigma-Aldrich	Cat# H7425
Anti-Protein A	Sigma-Aldrich	Cat# P3775
Anti-HA	Abcam	Cat# ab18181
RPL5 Antibody	Cell Signaling Technology	Cat# 14568
**Chaetomium thermophilum strains**		ID in Hurt Lab spores collection
C. thermophilum: Erg1::P_{ACTIN}-pA-TEV-FLAG-*ct*RPF2 (CTHT_0061110)	This study	*ct*Rpf2-FtpA
C. thermophilum: Erg1::P_{ACTIN}-pA-TEV-FLAG-*ct*RRS1 (CTHT_0057260)	This study	*ct*Rrs1-FtpA
C. thermophilum: Erg1::P_{ACTIN}-pA-TEV-FLAG-*ct*SYO1 (CTHT_0033460)	This study	*ct*Syo1-FtpA
**Yeast strains**		ID in Hurt Lab yeast collection
**w303** wild type (*S. cerevisiae*)	[1]	Y-3609
syo1Δ:<br>w303: SYO1::natNT2	[2]	Y-3833
syo1Δ, rpl5Δ shuffle strain:<br>w303: SYO1::natNT2; RPL5::His3MX6, pRS316-*sc*UL18	[2]	Y-4575
rpf2Δ shuffle strain:<br>w303: RPF2::natNT2; pRS316-RPF2	This study	Y-5697
rrs1Δ shuffle strain:<br>w303: RRS1::natNT2, pRS316-RRS1	This study	Y-5693
syo1Δ, rpf2Δ shuffle strain:<br>w303: SYO1::natNT2, RPF2::His3MX6, pRS316-RPF2	This study	Y-6381
**Nsa1-FtpA**:<br>w303: NSA1-FtpA:natNT2	This study	Y-6382
**Nsa3-FtpA**:<br>w303: NSA3-FtpA:natNT2	This study	Y-6383
**Yvh1-FtpA**:<br>w303: YVH1-FtpA:klURA3	This study	Y-5560
**Nsa1-FtpA, GAL::HA-Rpf2**:<br>w303: NSA1-FtpA:natNT2, His3MX6:P_{GAL}-HA-RPF2	This study	Y-6384
**Nsa1-FtpA, GAL::HA-Rrs1**:<br>w303: NSA1-FtpA:natNT2, His3MX6:P_{GAL}-HA-RRS1	This study	Y-6385

**Nsa1-FtpA, GAL::HA-uL18**:<br>w303: NSA1-FtpA:natNT2, His3MX6:P_{GAL}-HA-UL18	This study	Y-6386
**Nsa3-FtpA, GAL::HA-Rpf2**:<br>w303: NSA3-FtpA:natNT2, His3MX6:P_{GAL}-HA-RPF2	This study	Y-6387
**Nsa3-FtpA, GAL::HA-Rrs1**:<br>w303: NSA3-FtpA:natNT2, His3MX6:P_{GAL}-HA-RRS1	This study	Y-6388
**Nsa3-FtpA, GAL::HA-uL18**:<br>w303: NSA3-FtpA:natNT2, His3MX6:P_{GAL}-HA-UL18	This study	Y-6389
**GAL::HA-Rpf2, GAL:HA-Rrs1**:<br>w303: His3MX6:P_{GAL}-HA-RPF2, His3MX6:P_{GAL}-HA-RRS1	This study	Y-6390
**PJ69-4**:<br>Strain for yeast two-hybrid assays	[3]	Y-4969
**Plasmids**		ID in Hurt Lab plasmid collection
Expression plasmid as negative control **YEplac112**<br>*2μ replication origin, TRP1 marker*	[4]	#550
Expression plasmid as negative control **YEplac181**<br>*2μ replication origin, LEU2 marker*	[4]	#552
Expression plasmid as negative control **YEplac195**<br>*2μ replication origin, URA3 marker*	[4]	#551
Expression plasmid as negative control **YCplac22**<br>*CEN4 (centromer vector), TRP1 marker*	[4]	#547
Expression plasmid as negative control **YCplac111**<br>*CEN4 (centromer vector), LEU2 marker*	[4]	#4549
Co-expression of ***ct*Rpf2** and **FLAG-*ct*Rrs1**:<br>pYEplac112-*ct*RPF2-$P_{GAL1/10}$-FLAG-ctRRS1	This study	#6884
Expression of **pA-TEV-*ct*Syo1**:<br>pYEplac181-$P_{GAL1/10}$-ProteinA-TEV-*ct*SYO1	This study	#6885
Co-expression of ***sc*Rpf2** and **FLAG-*sc*Rrs1**:<br>pYEplac112-*sc*Rpf2-$P_{GAL1/10}$-FLAG-*sc*Rrs1	This study	#6886
Expression of **pA-TEV-*sc*Syo1**:<br>pYEplac181-$P_{GAL1/10}$-ProteinA-TEV-*sc*Syo1	This study	#6887
Plasmid for co-expression of ***sc*uL18** and ***sc*uL5**:<br>pYEplac195-*sc*UL18-$P_{GAL1/10}$-*sc*UL5	This study	#6888
Plasmid for co-expression of ***sc*uL18** and **pA-TEV-*sc*uL5**:<br>pYEplac195-*sc*UL18-$P_{GAL1/10}$-ProteinA-TEV-*sc*UL5	This study	#6889
Plasmid for expression of ***sc*Syo1**:<br>pYEplac181-$P_{GAL1/10}$-*sc*SYO1	This study	#6890
Plasmid for co-expression of ***sc*Rpf2ΔC** and **FLAG-*sc*Rrs1ΔC**:<br>pYEplac112-*sc*RPF2(1-252aa)-$P_{GAL1/10}$-FLAG-*sc*RRS1(1-108 aa)	This study	#6891
Plasmid for co-expression of ***sc*uL18-HA** and ***sc*uL5-3xGFP**:<br>pYEplac195-*sc*UL18-HA-$P_{GAL1/10}$-*sc*UL5-(3x)yeGFP	This study	#6892
Plasmid for expression of **pA-TEV-Syo1-HA**:<br>pYEplac181-$P_{GAL1/10}$-ProteinA-TEV-*sc*SYO1-HA	This study	#6893
Plasmid for co-expression of ***sc*Rpf2-HA** and **FLAG-HA-*sc*Rrs1**:<br>pYEplac112-*sc*RPF2-HA-$P_{GAL1/10}$-FLAG-HA-*sc*RRS1	This study	#6894
Plasmid for co-expression of ***sc*uL5-HA** and ***sc*uL18-HA**:<br>pYCplac195-*sc*UL5-HA-$P_{GAL1/10}$-*sc*UL18-HA	This study	#6895

Plasmid for co-expression of ***hs*Rrs1** and **FLAG-*hs*Rpf2**:<br>pYCplac112-*hs*RRS1-$P_{GAL1/10}$-FLAG-*hs*RPF2	This study	#6896
Plasmid for co-expression of **hsuL18** and **pA-TEV-hsuL5**:<br>pYCplac181-*hs*UL18-$P_{GAL1/10}$-ProteinA-TEV-*hs*UL5	This study	#6897
Plasmid for expression of ***hs*Syo1**:<br>pYCplac195-PGAL1/10-HEATR3	This study	P #6898
Plasmid for expression of ***sc*Rpf2-FtpA**:<br>pYCplac111-P_{RPF2}-*sc*RPF2-FLAG-TEV-ProteinA	This study	#5785
Plasmid for expression of ***hs*Rpf2-FtpA**:<br>pYCplac111-P_{RPF2}-*hs*RPF2-FLAG-TEV-ProteinA	This study	#6899
Plasmid for expression of ***sc*Rpf2-FtpA** (*in vivo* binding assay):<br>pYCplac111-P_{RPF2}-*sc*RPF2-FLAG-TEV-ProteinA	This study	#5785
Plasmid for expression of ***sc*Rpf2ΔC-FtpA** (*in vivo* binding assay):<br>pYCplac111-P_{RPF2}-*scRPF2*(1-252 aa)-FLAG-TEV-ProteinA	This study	#5805
Plasmid for expression of ***sc*Rrs1** (*in vivo* binding assay):<br>pYCplac22-P_{RRS1}-*sc*RRS1	This study	#6900
Plasmid for expression of ***sc*Rrs1ΔC** (*in vivo* binding assay):<br>pYCplac22-P_{RRS1}-*scRRS1*(1-108 aa)	This study	#6901
Plasmid for expression of ***sc*Rpf2** (complementation assays):<br>pYCplac111-P_{RPF2}-*sc*RPF2	This study	#6902
Plasmid for expression of ***sc*Rpf2ΔC** (complementation assays):<br>pYCplac111-P_{RPF2}-*scRPF2*(1-252 aa)	This study	#6903
Plasmid for expression of ***sc*Rrs1** (complementation assays):<br>pYCplac111-P_{RRS1}-*sc*RRS1	This study	#5749
Plasmid for expression of ***sc*Rrs1ΔC** (complementation assays):<br>pYCplac111-P_{RRS1}-sc*RRS1*(1-108 aa)	This study	#6904
Plasmid for expression of ***hs*Rpf2** (complementation assays):<br>pYCplac111-P_{RPF2}-*hs*RPF2	This study	#6905
Plasmid for expression of ***ct*Rpf2** (complementation assays):<br>pYCplac111-P_{RPF2}-*ct*RPF2	This study	#6906
Plasmid for expression of ***hs*Rrs1** (complementation assays):<br>pYCplac111-P_{RRS1}-*hs*RRS1	This study	#6907
Plasmid for expression of ***ct*Rrs1** (complementation assays):<br>pYCplac111-P_{RRS1}-*ct*RRS1	This study	#6908
Plasmid for expression of ***sc*Rpf2** (over-expression assays):<br>pYEplac112-$P_{GAL1/10}$-*sc*RPF2	This study	#6909
Plasmid for expression of ***sc*Rpf2ΔC** (over-expression assays):<br>pYEplac112-$P_{GAL1/10}$-*scRPF2*(1-252 aa)	This study	#6910
Plasmid for expression of ***sc*Rrs1** (over-expression assays):<br>pYEplac112-$P_{GAL1/10}$-*sc*RRS1	This study	#5815
Plasmid for expression of ***sc*Rrs1ΔC** (over-expression assays):<br>pYEplac112-$P_{GAL1/10}$-*scRRS1*(1-108 aa)	This study	#5816
Plasmid for **25S rRNA(H81-H87)** *in vitro* synthesis (EMSA assays):<br>pBlueScript-P_{T7}-25SrRNA(H81-H87)	This study	#6911
Plasmid for **Phe-tRNA** *in vitro* synthesis (EMSA assays):<br>pBlueScript-P_{T7}-$tRNA^{PHE}$	This study	#512
Plasmid for expression of ***sc*uL18** (yeast growth analysis):<br>pYCplac111-P_{UL18}-*sc*UL18	This study	#6912
Plasmid for expression of ***sc*uL18 169G>S** (yeast growth analysis):<br>pYCplac111-P_{UL18}-*scUL18 (169 G>S)*	This study	#6913

Plasmid for expression of ***sc*Syo1** (yeast growth analysis):<br>pYCplac22-P_{SYO1}-*sc*SYO1	This study	#6914
Plasmid for expression of ***sc*Syo1-A** (yeast growth analysis):<br>pYCplac22-P_{SYO1}-*scSYO1 (118 K>E)*	This study	#6915
Plasmid for expression of ***sc*Syo1-B** (yeast growth analysis):<br>pYCplac22-P_{SYO1}-*scSYO1 (74K>E, 76K>E)*	This study	#6916
Plasmid for expression of ***sc*Syo1-AB** (yeast growth analysis):<br>pYCplac22-P_{SYO1}-*scSYO1 (74K>E, 76K>E, 118 K>E)*	This study	#6917
Plasmid for expression of **FLAG-*sc*uL18**:<br>pYEplac112-$P_{GAL1/10}$-FLAG-*sc*UL18	This study	#6918
Plasmid for expression of **FLAG-*sc*uL18 169G>S**:<br>pYEplac112-$P_{GAL1/10}$-FLAG-sc*UL18 (169G>S)*	This study	#6919
Plasmid for expression of **pA-TEV-*sc*Syo1-AB**:<br>pYEplac181-$P_{GAL1/10}$-ProteinA-TEV-*scSYO1 (74K>E, 76K>E, 118 K>E)*	This study	#6920
Plasmid for expression of ***hs*Syo1-FLAG**:<br>pYEplac181-$P_{GAL1/10}$-MDM2-FLAG	This study	#6921
Plasmid for co-expression of ***hs*uL5** and ***hs*uL18-TEV-pA**:<br>pYEplac112-*hs*UL5-$P_{GAL1/10}$-*hs*UL18-TEV-ProteinA	This study	#6922
Plasmid for expression of ***hs*uL18-TEV-pA (1-20 aa)**:<br>pYEplac112-$P_{GAL1/10}$-*hsUL18*(1-20 aa)-TEV-ProteinA	This study	#6936
Plasmid for expression of ***hs*uL18-TEV-pA (1-30 aa)**:<br>pYEplac112-$P_{GAL1/10}$-*hsUL18*(1-30 aa)-TEV-ProteinA	This study	#6923
Plasmid for expression of ***hs*uL18-TEV-pA (1-41 aa)**:<br>pYEplac112-$P_{GAL1/10}$-*hsUL18*(1-41 aa)-TEV-ProteinA	This study	#6924
Plasmid for expression of ***hs*uL18-TEV-pA (21-297 aa)**:<br>pYEplac112-$P_{GAL1/10}$-*hsUL18*(21-297 aa)-TEV-ProteinA	This study	#6925
Plasmid for expression of ***hs*uL18-TEV-pA (31-297 aa)**:<br>pYEplac112-$P_{GAL1/10}$-*hsUL18*(31-297 aa)-TEV-ProteinA	This study	#6926
Plasmid for expression of ***hs*uL18-TEV-pA (42-297 aa)**:<br>pYEplac112-$P_{GAL1/10}$-*hsUL18*(42-297 aa)-TEV-ProteinA	This study	#6927
Yeast two-hybrid control plasmid **pGADT7-SV40**:<br>LEU2 marker, T7 promoter, SV40-AD (Activation Domain)	[5]	#3724
Plasmid for expression of ***hs*uL18** (Y2H assay):<br>pGADT7-*hs*UL18-AD	This study	#6928
Plasmid for expression of ***hs*uL18 (1-20 aa)** (Y2H assay):<br>pGADT7-*hs*UL18(1-20 aa)-AD	This study	#6929
Plasmid for expression of ***hs*uL18 (1-30 aa)** (Y2H assay):<br>pGADT7-*hs*UL18(1-30 aa)-AD	This study	#6930
Plasmid for expression of ***hs*uL18 (1-41 aa)** (Y2H assay):<br>pGADT7-*hs*UL18(1-41 aa)-AD	This study	#6931
Plasmid for expression of ***hs*uL18 (21-297 aa)** (Y2H assay):<br>pGADT7-*hs*UL18(21-297 aa)-AD	This study	#6932
Plasmid for expression of ***hs*uL18 (31-297 aa)** (Y2H assay):<br>pGADT7-*hs*UL18(31-297 aa)-AD	This study	#6933
Plasmid for expression of ***hs*uL18 (22-297 aa)** (Y2H assay):<br>pGADT7-*hs*UL18(42-297 aa)-AD	This study	#6934
Yeast two-hybrid control plasmid **pGBKT7-p53**:<br>TRP1 marker, T7 promoter, p53-BD (Binding Domain)	[6]	#3467

Plasmid for expression of **MDM2** (Y2H assay): pGBKT7-MDM2-BD	This study	#6935
**Reagents and chemicals**		
3X FLAG® Peptide lyophilized powder	Sigma-Aldrich	Cat# F3290
TEV protease	Sigma-Aldrich	Cat# T4455
SIGMAFAST™ Protease inhibitor tablets for general use	Sigma-Aldrich	Cat# S8820
[alpha-P32] UTP, 3000 Ci/mmol, 10 mCi/ml	Hartman Analytic	Cat# SRP-210
IGEPAL® CA-630	Sigma-Aldrich	Cat# I8896
DSS-H12/D12	Creative Molecules	Cat# 001S
Coomassie Brilliant Blue R-250	Thermo Fisher Scientific	Cat# 20278
SYBR®Green II RNA gel stain	Sigma-Aldrich	Cat# S9305
ANTI-FLAG® M2 Affinity Gel	Sigma-Aldrich	Cat# A2220
IgG Sepharose® 6 Fast Flow	Sigma-Aldrich	Cat# GE17-0969-01
HiScribe-T7 High Yield RNA Synthesis Kit	NEB	Cat# E2040S
NuPAGE™ 4 to 12%, Bis-Tris	Thermo Fisher Scientific	Cat# NP0322PK2, Cat# WG1403A
**Oligonucleotides**		
S. cerevisiae 5S rRNA probe: 5'-CTACTCGGTCAGGCTC-3'	This study	N/A
C. thermophilum 5S rRNA probe: 5'-TCAGTGGCTTGTCTATGG-3'	This study	N/A
**Software and algorithms**		
MaxQuant software v1.6.17.0. https://www.maxquant.org	[7]	N/A
xQuest http://proteomics.ethz.ch	[8]	N/A
xProphet http://proteomics.ethz.ch	[9]	N/A

xiNet<br>http://crosslinkviewer.org/	10	N/A
Xlink Analyzer<br>https://www.embl-hamburg.de/XlinkAnalyzer/XlinkAnalyzer.html	11	N/A
UCSF Chimera<br>https://www.cgl.ucsf.edu/chimera/	12	N/A
UCSF ChimeraX<br>https://www.cgl.ucsf.edu/chimerax/	13	N/A
EMAN2<br>https://blake.bcm.edu/emanwiki/EMAN2	14	N/A
IMAGIC-4D<br>https://www.imagescience.de/imagic-4d.html	15	N/A
Gctf<br>https://www2.mrc-lmb.cam.ac.uk/research/locally-developed-software/zhang-software/	16	N/A
CTFFIND4<br>https://grigoriefflab.umassmed.edu/ctffind4	17	N/A
CrYOLO<br>https://cryolo.readthedocs.io/en/stable/	18	N/A
cryoSPARC<br>https://cryosparc.com/	19	N/A
Relion 3.1<br>https://relion.readthedocs.io/en/latest/	20,21	N/A
SWISS-MODEL<br>https://swissmodel.expasy.org	22	N/A
Coot<br>https://www2.mrc-lmb.cam.ac.uk/personal/pemsley/coot/	23	N/A
Phenix<br>http://www.phenix-online.org/	24,25	N/A

Supplemental references

1. Thomas, B. J. & Rothstein, R. Elevated recombination rates in transcriptionally active DNA. *Cell* **56**, 619–630 (1989).
2. Kressler, D. *et al.* Synchronizing Nuclear Import of Ribosomal Proteins with Ribosome Assembly. *Science* **338**, 666–671 (2012).
3. James, P., Halladay, J. & Craig, E. A. Genomic Libraries and a Host Strain Designed for Highly Efficient Two-Hybrid Selection in Yeast. *Genetics* **144**, 1425–1436 (1996).

4. Gietz, R. D. & Sugino, A. New yeast-Escherichia coli shuttle vectors constructed with in vitro mutagenized yeast genes lacking six-base pair restriction sites. *Gene* **74**, 527–534 (1988).

5. Chien, C. T., Bartel, P. L., Sternglanz, R. & Fields, S. The two-hybrid system: a method to identify and clone genes for proteins that interact with a protein of interest. *Proc Natl Acad Sci U S A* **88**, 9578–9582 (1991).

6. Luo, Y., Batalao, A., Zhou, H. & Zhu, L. Mammalian two-hybrid system: a complementary approach to the yeast two-hybrid system. *Biotechniques* **22**, 350–352 (1997).

7. Luber, C. A. *et al.* Quantitative Proteomics Reveals Subset-Specific Viral Recognition in Dendritic Cells. *Immunity* **32**, 279–289 (2010).

8. Leitner, A., Walzthoeni, T. & Aebersold, R. Lysine-specific chemical cross-linking of protein complexes and identification of cross-linking sites using LC-MS/MS and the xQuest/xProphet software pipeline. *Nature Protocols* **9**, 120–137 (2014).

9. Walzthoeni, T. *et al.* False discovery rate estimation for cross-linked peptides identified by mass spectrometry. *Nature Methods* **9**, 901–903 (2012).

10. Combe, C. W., Fischer, L. & Rappsilber, J. xiNET: Cross-link Network Maps With Residue Resolution. *Mol Cell Proteomics* **14**, 1137–1147 (2015).

11. Kosinski, J. *et al.* Xlink Analyzer: Software for analysis and visualization of cross-linking data in the context of three-dimensional structures. *J Struct Biol* **189**, 177–183 (2015).

12. Pettersen, E. F. *et al.* UCSF Chimera—A visualization system for exploratory research and analysis. *Journal of Computational Chemistry* **25**, 1605–1612 (2004).

13. Goddard, T. D. *et al.* UCSF ChimeraX: Meeting modern challenges in visualization and analysis. *Protein Sci* **27**, 14–25 (2018).

14. Tang, G. *et al.* EMAN2: an extensible image processing suite for electron microscopy. *J Struct Biol* **157**, 38–46 (2007).

15. Heel, M. van *et al.* Four-dimensional cryo-electron microscopy at quasi-atomic resolution: IMAGIC 4D. in *International Tables for Crystallography* 624–628 (American Cancer Society, 2012). doi:10.1107/97809553602060000875.

16. Zhang, K. Gctf: Real-time CTF determination and correction. *J Struct Biol* **193**, 1–12 (2016).

17. Rohou, A. & Grigorieff, N. CTFFIND4: Fast and accurate defocus estimation from electron micrographs. *J Struct Biol* **192**, 216–221 (2015).

18. Wagner, T. *et al.* SPHIRE-crYOLO is a fast and accurate fully automated particle picker for cryo-EM. *Commun Biol* **2**, 218 (2019).

19. Punjani, A., Rubinstein, J. L., Fleet, D. J. & Brubaker, M. A. cryoSPARC: algorithms for rapid unsupervised cryo-EM structure determination. *Nat Methods* **14**, 290–296 (2017).

20. Zivanov, J. *et al.* New tools for automated high-resolution cryo-EM structure determination in RELION-3. *Elife* **7**, (2018).

21. Zivanov, J., Nakane, T. & Scheres, S. H. W. Estimation of high-order aberrations and anisotropic magnification from cryo-EM data sets in RELION-3.1. *IUCrJ* **7**, 253–267 (2020).

22. Waterhouse, A. *et al.* SWISS-MODEL: homology modelling of protein structures and complexes. *Nucleic Acids Res* **46**, W296–W303 (2018).

23. Emsley, P. & Cowtan, K. Coot: model-building tools for molecular graphics. *Acta Crystallogr D Biol Crystallogr* **60**, 2126–2132 (2004).

24. Adams, P. D. *et al.* PHENIX: a comprehensive Python-based system for macromolecular structure solution. *Acta Crystallogr D Biol Crystallogr* **66**, 213–221 (2010).

25. Liebschner, D. *et al.* Macromolecular structure determination using X-rays, neutrons and electrons: recent developments in Phenix. *Acta Cryst D* **75**, 861–877 (2019).
