# Supplementary figures and images for: Structure of nascent 5S RNPs at the crossroad between ribosome assembly and MDM2–p53 pathways
Source: Nat Struct Mol Biol. 2023 Jun 8;30(8):1119–31. doi: 10.1038/s41594-023-01006-7 (PMC10442235; doi:10.1038/s41594-023-01006-7)

Probe: sc5S rRNA  
(5'-CTACTCGGTCAGGCTC-3')

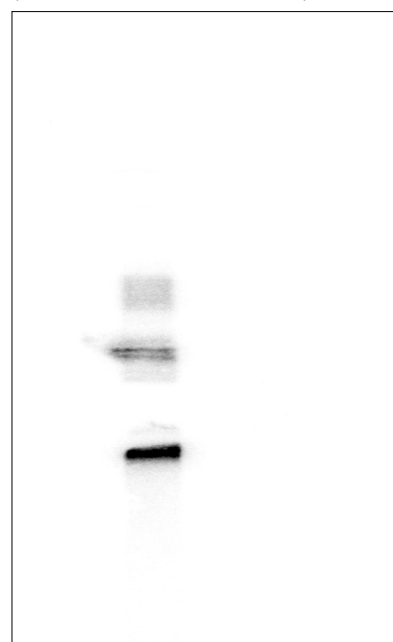

— 100 nt

— 100 nt

Supplement: Source Data Fig. 1 — Northern blots, uncropped images. [file 41594_2023_1006_MOESM5_ESM.pdf]

Fig. 4c - Northern blots

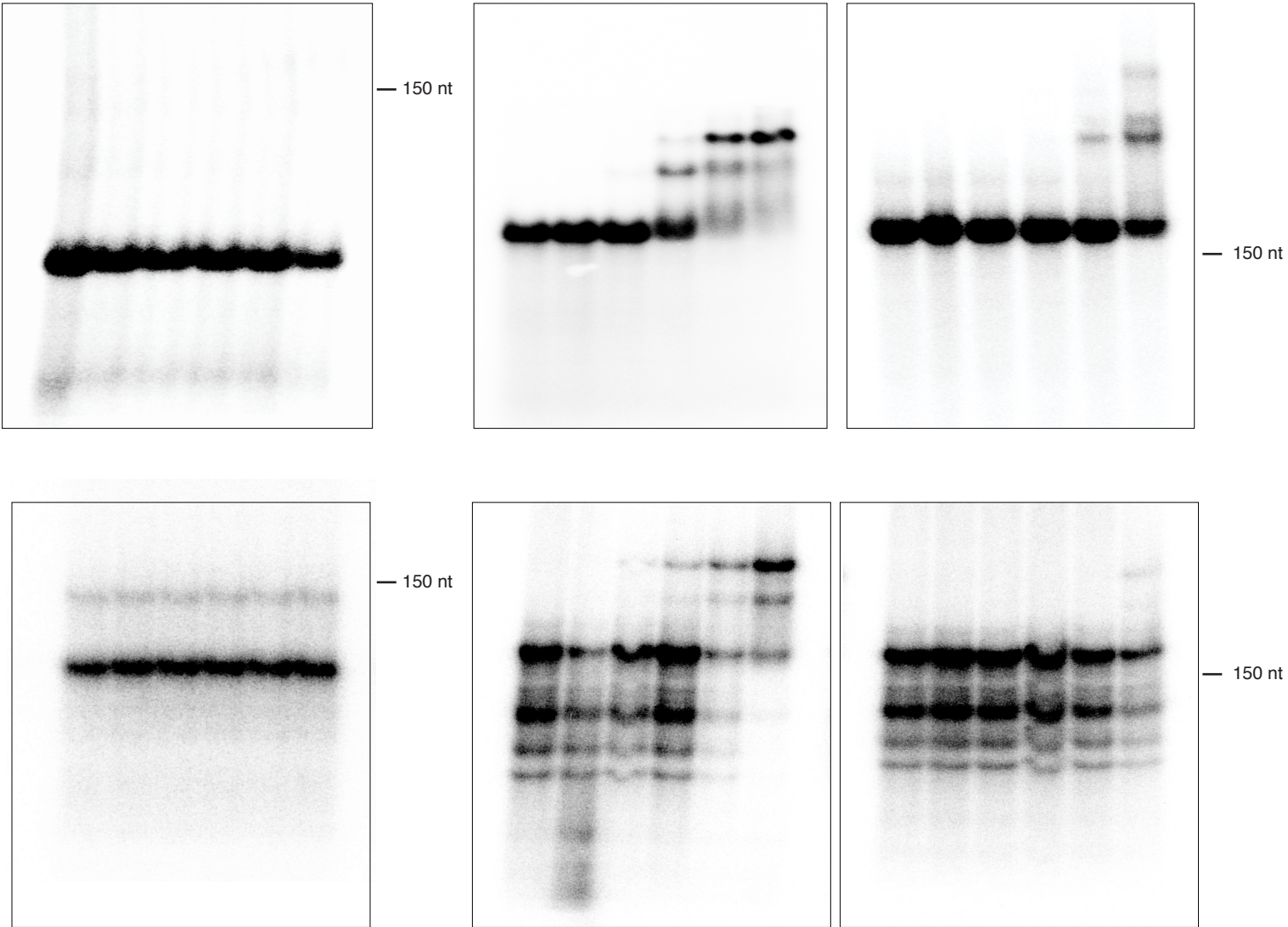

Supplement: Source Data Fig. 4 — Northern blots, uncropped images. [file 41594_2023_1006_MOESM6_ESM.pdf]

**Fig. 5a - Bottom panel (Methylene Blue Staining)**

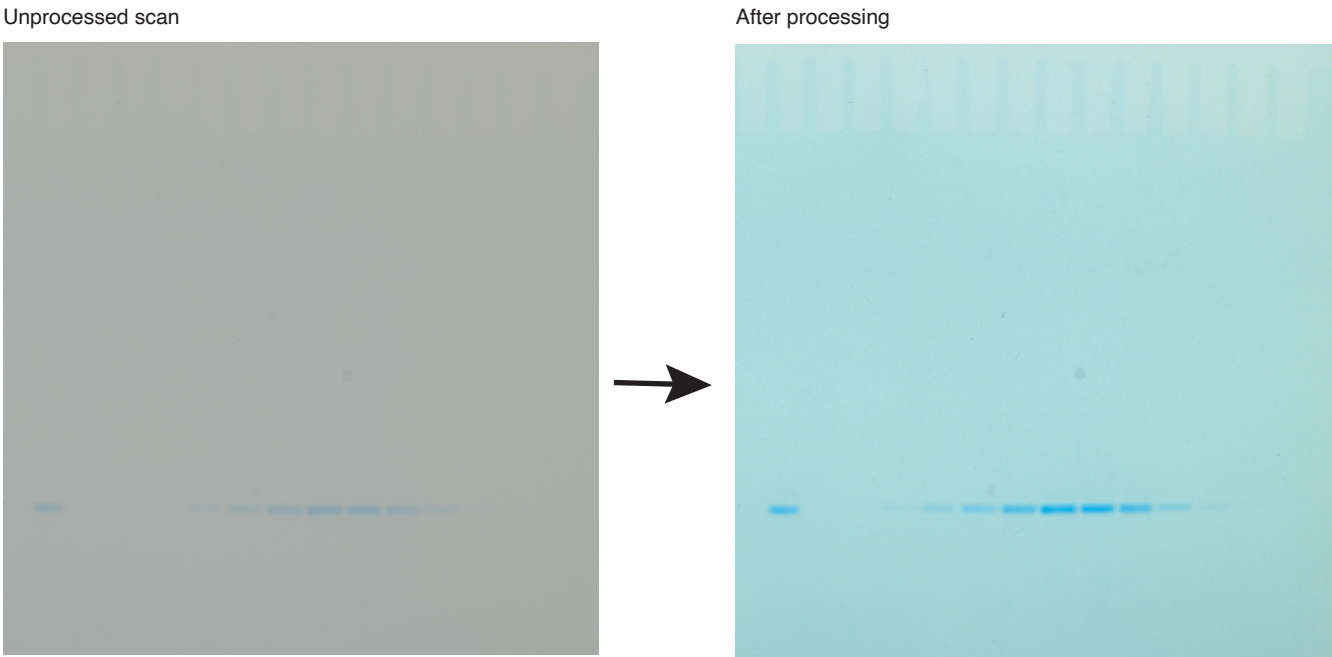

Supplement: Source Data Fig. 5 — Methylene blue staining, uncropped images. [file 41594_2023_1006_MOESM7_ESM.pdf]

**Fig. 6c - Middle bottom panel (Western Blot)**

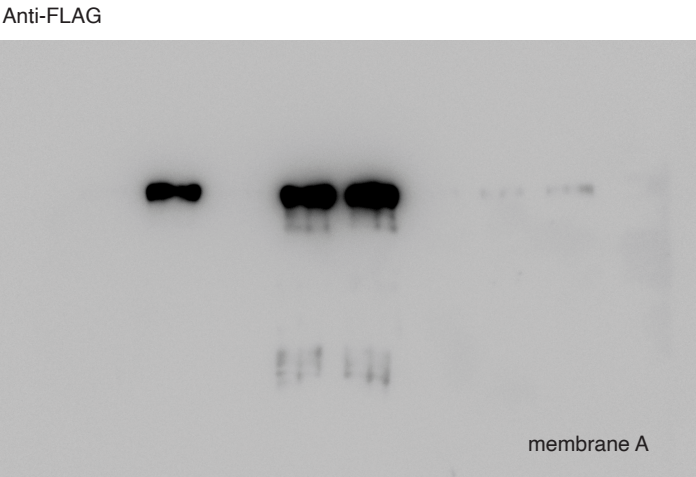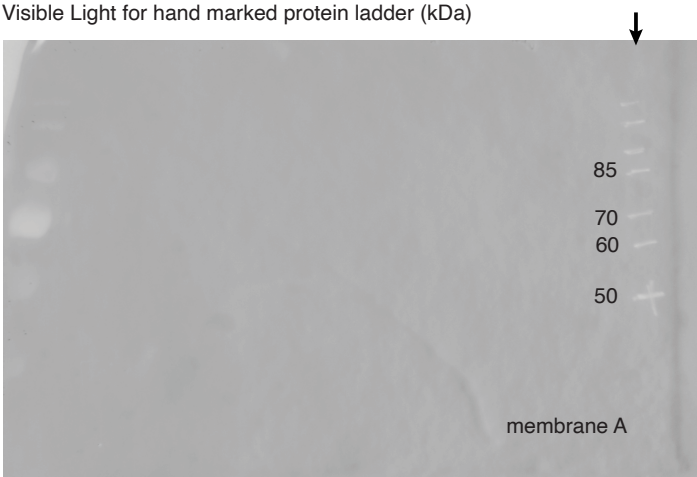

**Fig. 6d - Bottom panels (Methylene Blue Staining)**

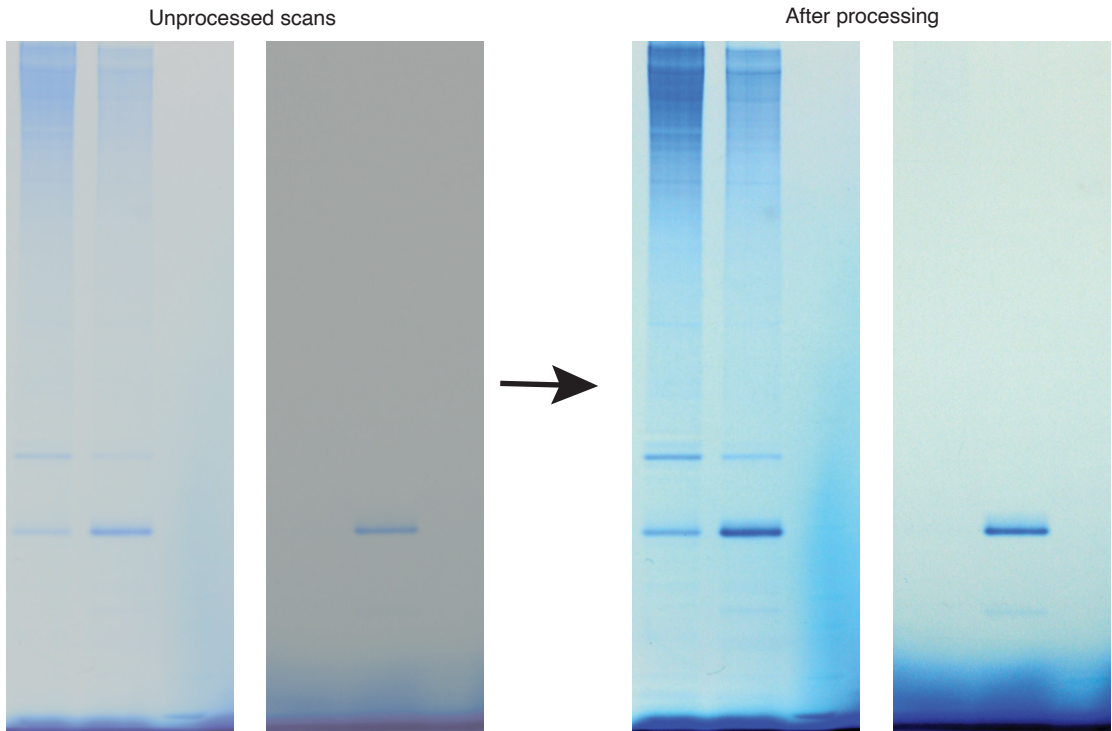

Supplement: Source Data Fig. 6 — Western blot, methylene blue staining and uncropped images. [file 41594_2023_1006_MOESM8_ESM.pdf]

Extended Data Figure 1a (Methylene Blue Staining)

Unprocessed scans

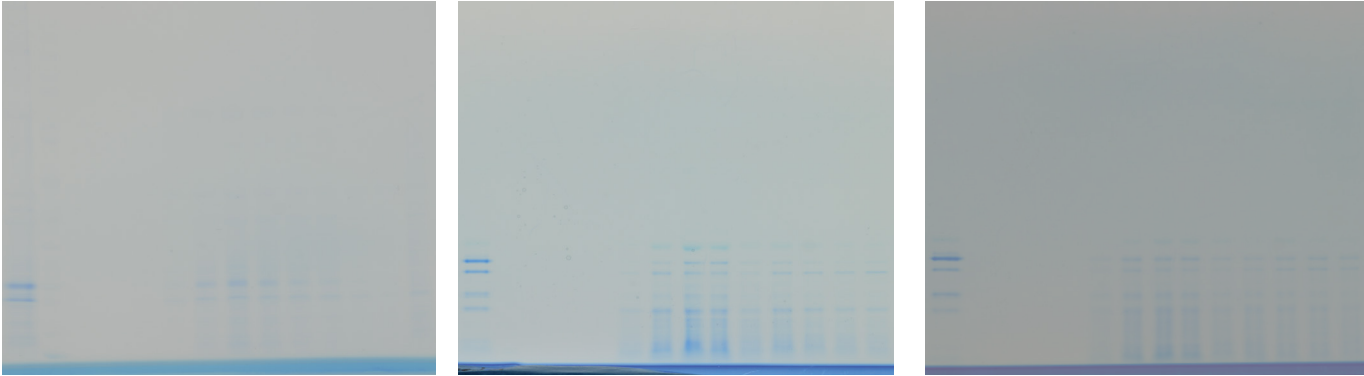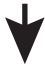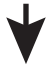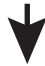

After processing

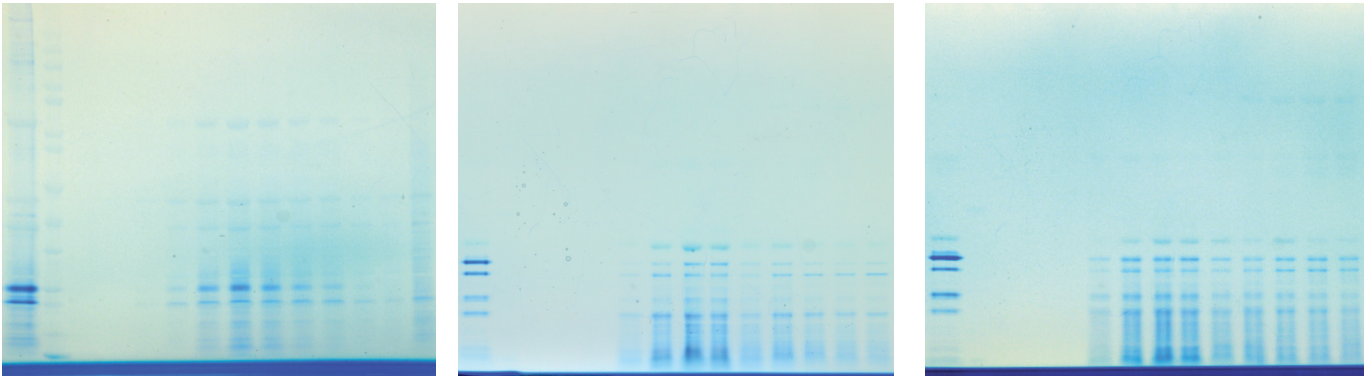

Supplement: Source Data Extended Data Fig. 1 — Methylene blue staining, uncropped images. [file 41594_2023_1006_MOESM9_ESM.pdf]

Extended Data Figure 4e (Methylene Blue Staining)

Unprocessed scan

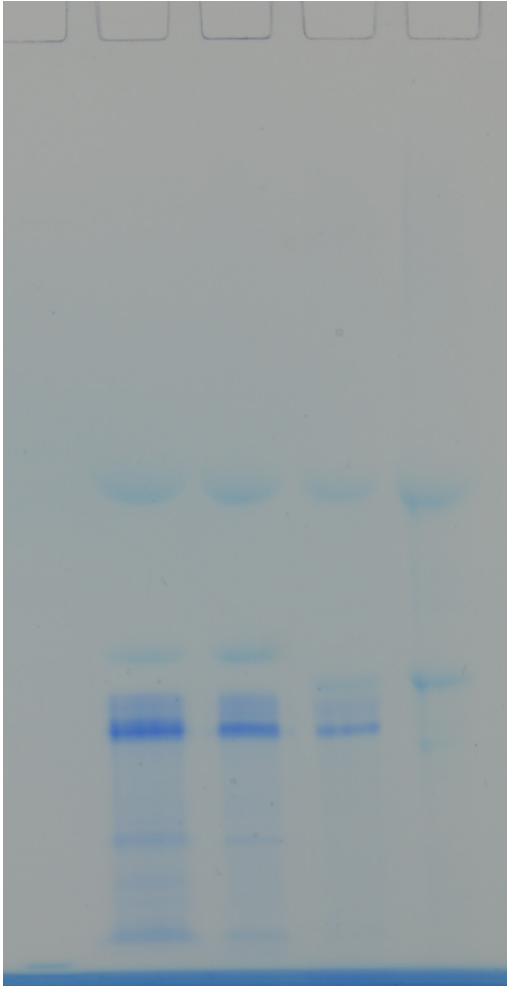

After processing

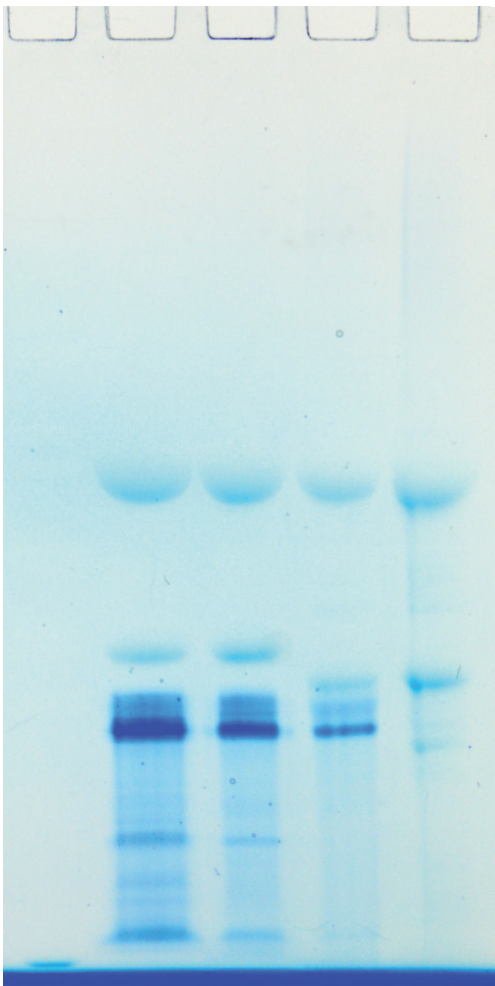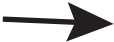

Supplement: Source Data Extended Data Fig. 4 — Methylene blue staining, uncropped images. [file 41594_2023_1006_MOESM10_ESM.pdf]
