# Supplementary material for: Structure of nascent 5S RNPs at the crossroad between ribosome assembly and MDM2–p53 pathways
Source: Nat Struct Mol Biol. 2023 Jun 8;30(8):1119–31. doi: 10.1038/s41594-023-01006-7 (PMC10442235; doi:10.1038/s41594-023-01006-7)

Extended Data Fig. 5b - Western blots

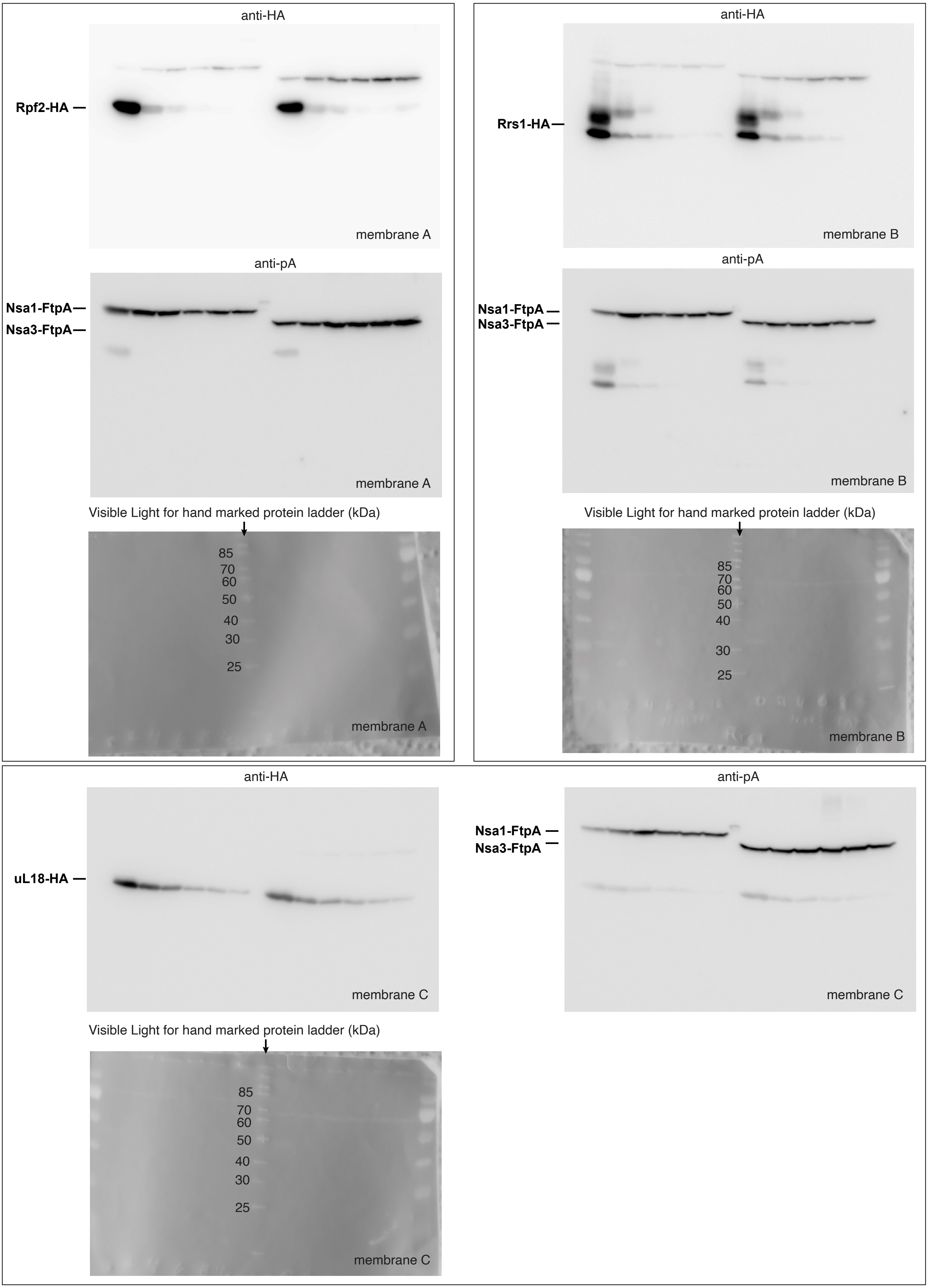

Extended Data Fig. 5c - Bottom panels (Methylene Blue Staining and Western blot)

Nsa1-FtpA

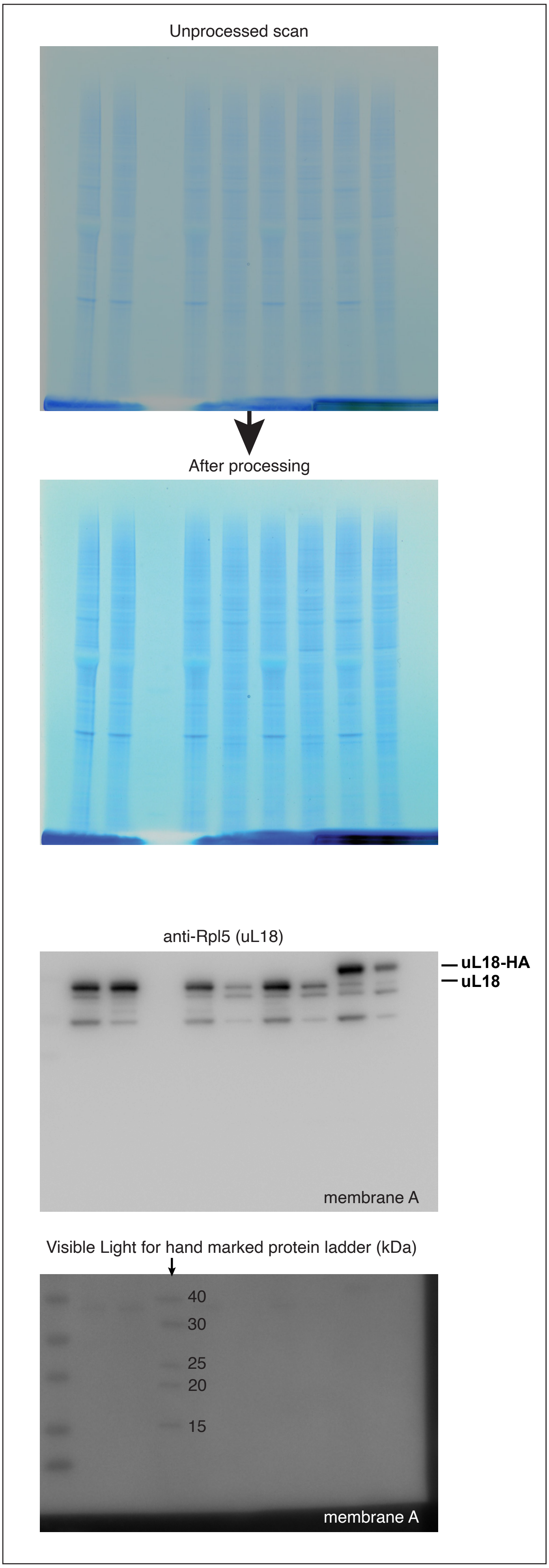

Nsa3-FtpA

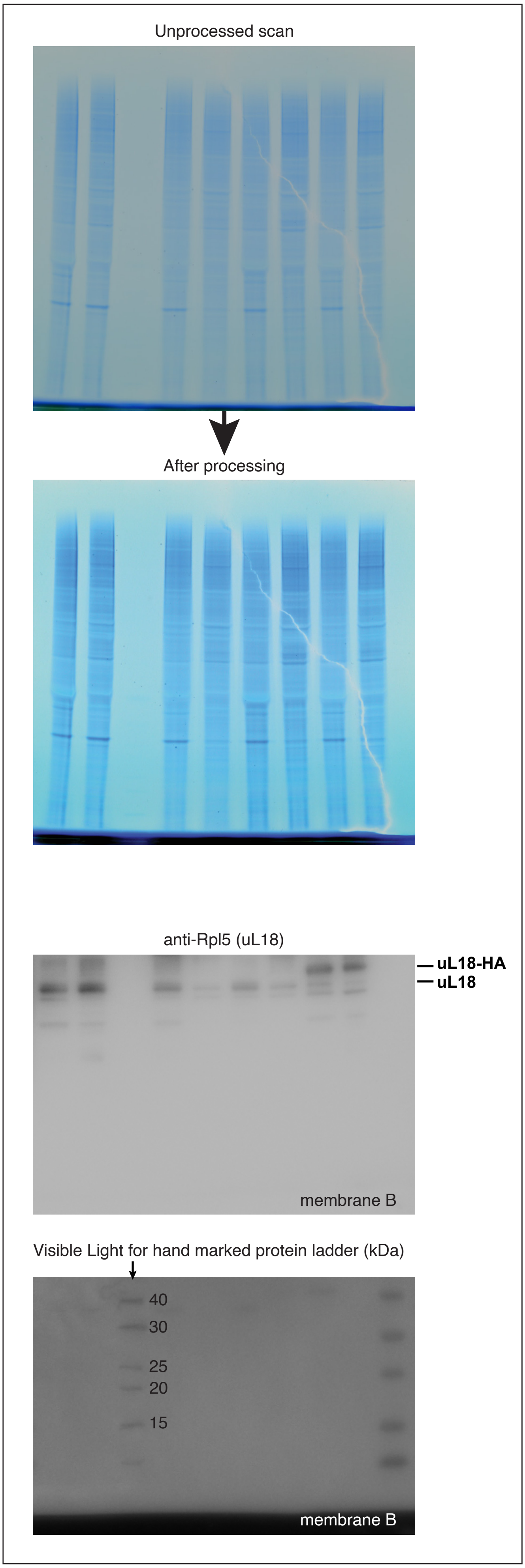

Supplement: Source Data Extended Data Fig. 5 — Methylene blue staining, western blot and uncropped images. [file 41594_2023_1006_MOESM11_ESM.pdf]
